# Unravelling the physicochemical and antimicrobial mechanisms of human serum albumin/tannic acid coatings for medical-grade polycaprolactone scaffolds

**DOI:** 10.1016/j.bioactmat.2024.08.023

**Published:** 2024-08-28

**Authors:** Silvia Cometta, Bogdan C. Donose, Alfredo Juárez-Saldivar, Akhilandeshwari Ravichandran, Yanan Xu, Nathalie Bock, Tim R. Dargaville, Aleksandar D. Rakić, Dietmar W. Hutmacher

**Affiliations:** a Faculty of Engineering, School of Mechanical, Medical and Process Engineering, Queensland University of Technology, Brisbane, QLD, 4000, Australia; b Australian Research Council Training Centre for Multiscale 3D Imaging, Modelling and Manufacturing (M3D Innovation), Queensland University of Technology, Kelvin Grove, QLD, 4059, Australia; c Max Planck Queensland Centre, Queensland University of Technology, Brisbane, QLD, 4000, Australia; d School of Electrical Engineering and Computer Science, The University of Queensland, Brisbane, QLD, 4072, Australia; e Unidad Académica Multidisciplinaria Reynosa Aztlán, Universidad Autónoma de Tamaulipas, Reynosa, 88740, Mexico; f Central Analytical Research Facility (CARF), Queensland University of Technology, Brisbane, QLD, 4000, Australia; g Faculty of Health, School of Biomedical Sciences, Queensland University of Technology, Brisbane, QLD, 4000, Australia; h Translational Research Institute, Woolloongabba, QLD, 4102, Australia; i Centre for Materials Science, School of Chemistry and Physics, Queensland University of Technology, Brisbane, QLD, 4000, Australia; j Australian Research Council Training Centre for Cell and Tissue Engineering Technologies, Queensland University of Technology, Brisbane, QLD, 4059, Australia

**Keywords:** Bacterial infection, Biofilm, Human serum albumin, Tannic acid, Polycaprolactone, 3D printing, Scaffold

## Abstract

Biofilm-related biomaterial infections are notoriously challenging to treat and can lead to chronic infection and persisting inflammation. To date, a large body of research can be reviewed for coatings which potentially prevent bacterial infection while promoting implant integration. Yet only a very small number has been translated from bench to bedside. This study provides an in-depth analysis of the stability, antibacterial mechanism, and biocompatibility of medical grade polycaprolactone (mPCL), coated with human serum albumin (HSA), the most abundant protein in blood plasma, and tannic acid (TA), a natural polyphenol with antibacterial properties. Molecular docking studies demonstrated that HSA and TA interact mainly through hydrogen-bonding, ionic and hydrophobic interactions, leading to smooth and regular assemblies. *In vitro* bacteria adhesion testing showed that coated scaffolds maintained their antimicrobial properties over 3 days by significantly reducing *S. aureus* colonization and biofilm formation. Notably, amplitude modulation-frequency modulation (AMFM) based viscoelasticity mapping and transmission electron microscopy (TEM) data suggested that HSA/TA-coatings cause morphological and mechanical changes on the outer cell membrane of *S. aureus* leading to membrane disruption and cell death while proving non-toxic to human primary cells. These results support this antibiotic-free approach as an effective and biocompatible strategy to prevent biofilm-related biomaterial infections.

## Highlights

• Enhanced antimicrobial activity - Scaffolds coated with HSA/TA are effective against microbial growth for up to three days, which significantly reduces the colonization of *S. aureus* and prevents biofilm formation.
• Bacterial membrane disruption – Coated scaffolds cause morphological and mechanical alterations in *S. aureus* cell membranes, leading to membrane disruption and cell death.
• Reinforced mechanical stability – Compared to their uncoated counterparts, mPCL scaffolds coated with HSA/TA demonstrated superior mechanical properties, which are crucial for their successful application as antibacterial implant coatings.
• Biocompatible design - Coated scaffolds did not show any signs of cytotoxicity on *in vitro* cultured human cell sheets.

## Graphical abstract

**Figure:**
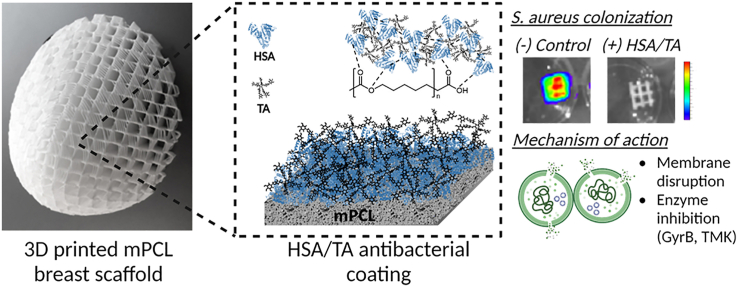

## 1 Introduction

During or immediately after surgery, a certain percentage of implants become contaminated with bacteria. Studies show that this percentage can range from as low as 0.1 % to as high as 20 %, on average [1,2]. Once in contact with the implant surface, bacteria can rapidly attach, aggregate, and form a protective biofilm which helps bacteria evade the endogenous defence system of the host, as well as external insults such as antibiotic use [3]. Biofilm-related implant infections are notoriously challenging to treat as the presence of a biofilm causes persisting inflammation of surrounding tissues that has been directly implicated in the pathogenesis of bone resorption, peri-implantitis, capsular contracture, and breast implant-associated anaplastic large cell lymphoma, among others [[4], [5], [6], [7]].

The gold standard method to prevent bacterial infection of implants is the systemic preoperative/operative administration of high dose antibiotics, as well as the surgical site irrigation with a triple-antibiotic solution [8]. Nevertheless, conventional administration of antibiotics is related to high toxicity, and several side effects to the patient [9]. Furthermore, the rapidly growing emergence of antibiotic-resistant strains has further impaired the efficacy of this approach [10].

Original strategies to prevent bacterial colonization and biofilm formation include tailoring the antibacterial properties of the implant surface by immobilizing alternative antimicrobial agents with improved spectrum of activity as well as the ability to combat multidrug-resistant bacteria. Despite the large amount of newly developed antimicrobial surfaces reported in the literature, only a few coatings have progressed to clinical trials, and even fewer to regulatory approval and clinical practice. The low translatability of these newly developed antibacterial surfaces can be attributed partly to the complexity of the problem, the lack of incorporation of realistic *in vivo* conditions when testing their efficacy, as well as the expensive and intricate regulatory procedures [11,12].

The modified surface should satisfy several safety, effectiveness, and economic criteria for a functionalized biomaterial surface to reach clinical applications. Notably, the coating/surface modification should possess adequate chemical and mechanical stability and adherence to the biomaterial surface to resist repeated mechanical loading during implantation; local toxicity should be minimal; modified biomaterials should possess high tissue biocompatibility, promoting implant integration; loaded antimicrobial agents should have a wide-spectrum activity as well as effectiveness against biofilm formation without generating antimicrobial resistance; finally, the process of surface modification should be affordable and technically simple to facilitate scalability for clinical translation [13,14]. Although significant progress has been made in modifying surfaces, no current approach meets all required criteria.

It has been demonstrated previously how using a combination of human serum albumin (HSA) and tannic acid (TA), could be effective in preventing *S. aureus* adhesion, one of the most commonly found bacteria in implant-related infection [15]. This study investigates the underlying mechanism of the molecular interactions between HSA and TA. In addition, we characterize in detail the topography, chemical composition, mechanical properties, antimicrobial mechanism, and *in vitro* biocompatibility of 3D printed medical grade polycaprolactone (mPCL) scaffolds with multiphasic porosity (from macro-to micro-to nanoscale), coated with 1%HSA/10%TA and 5%HSA/1%TA.

## 2 Materials and methods

### 2.1 Materials

Lyophilized HSA (≥96 %), TA (ACS grade), sucrose (≥99.5 %) were purchased from Merck (Germany). Gram-positive *Staphylococcus aureus* (*S. aureus* ATCC 29213) was procured from In Vitro Technologies (Victoria, Australia). *Staphylococcus aureus* Xen 36 and XenoLight D-Luciferin Potassium Salt were purchased from PerkinElmer (USA). QCM 5 MHz 14 mm Cr/Au sensors were obtained from QuartzPro (Sweden). Luria-Bertani (LB) broth and agar were acquired from Thermo Fisher Scientific (USA). All chemicals were purchased from Merck (Germany) unless specified otherwise.

### 2.2 Methods

#### 2.2.1 Scaffold fabrication

Macroporous scaffolds of a medical grade PCL combined with 45 % (w/w) of sugar particles with crystals size ranging from 20 to 50 μm, were fabricated using a 3D printer BioScaffolder 3.1 (GeSiM mbH, Germany). The printed scaffolds were immersed in ultrapurified water (Arium® pro UF Ultrapure Water System, Germany) for 15 days to leach out the sugar particles and create microporosity on the surface and within the scaffold struts. Fabricated scaffolds were plasma treated using a vacuum plasma cleaner (PDC-002-HP Harrick Plasma, USA) under O_2_/Ar_2_ for 4 min at medium (38W) power and subsequently sterilized by exposure to 70 % ethanol (% v/v) followed by evaporation. Scaffolds were then incubated in 1 % HSA and 5 % HSA solutions overnight, at room temperature and under agitation. Resulting layers of 1 % HSA and 5 % HSA were subsequently stabilized/crosslinked by incubating the HSA-coated scaffolds with 10%TA and 1%TA respectively, as described previously [15].

#### 2.2.2 Molecular docking

To study the binding mechanism between HSA and TA, crystal structure of HSA was retrieved from the RCSB protein data bank (PDB) (PDB ID: 1BM0, www.rcsb.org). Structure preparation was carried out with the Dock Prep tool in UCSF Chimera [16], where non protein residues along with water molecules were removed, sidechains were repaired, hydrogens were added, and partial charges were assigned. Later, the 3D coordinates of TA structure were obtained through ChemSpider (http://www.chemspider.com/Chemical-Structure.17286569.html). Finally, the molecular docking between HSA and TA was performed with gnina [17]. For the search space, the entire surface of the protein was considered. The docking tool generates nine conformations scored using free energy estimations and a convolutional neural network model that predicts the most favorable poses. To have a better description of the complexes generated by docking the non-covalent interactions were calculated with ProLIF [18].

To study the interactions between TA and different *S. aureus* targets, the 3D structures of DNA gyrase subunit B, dihydrofolate reductase, enoyl-[acyl-carrier-protein] reductase, and thymidylate kinase were retrieved from the RCSB PDB (www.rcsb.org) using the IDs: 4URM, 3FRF, 3GR6 and 4QGG, respectively. Subsequently, a molecular docking analysis was performed to compare the interaction of TA with each protein structure, alongside other known inhibitors of these proteins using GNINA 1.0 [17]. LigPlot + v.2.2 [19] was used to plot in 2D diagrams the interaction in each complex. The protein ligand interaction profiler (PLIP) [20] was used to calculate the interaction profile of each docking structure.

Finally, a structure-based study was used in order to assess the potential interactions of TA with known drug targets against *S. aureus*. First, four PDB structures containing known *S. aureus* drug targets in complex with an inhibitor were used to calculate the non-covalent interactions involved in the binding mode of each. Later, the estimation of free energy of binding and non-covalent interactions of TA with those drug targets were calculated by molecular docking.

#### 2.2.3 Molecular dynamics

To analyze the stability of the HSA-TA docked complex, a 100-ns MD simulation was performed using OpenMM using the most favorable conformation of TA obtained from docking. First, protein and ligand topologies were generated using force fields ff19SB and GAFF2, respectively. Later, the simulation box of 12 Å was filled with a TIP3P water model and neutralized with Na^+^ and Cl^−^. This system was minimized with a tolerance of 10 kJ/mol. Then, a 5-ns equilibration stage was implemented in the NPT ensemble at 300 K and 1 atm with a Monte Carlo Barostat and the Langevin Integrator. For the MD simulation the non-bounded interactions were calculated with Particle Mesh-Ewald using a cutoff of 1 nm. To analyze the trajectory, ProLIF was used to calculate the relative frequency of interactions in the last 50-ns trajectory and pytraj was used to quantify the strength of these interactions using the module for energy decomposition based on the linear interaction energy.

#### 2.2.4 Fourier transform infrared nanospectroscopy (nano-FTIR)

Nano-FTIR based on a scattering type scanning near-field optical microscope (s-SNOM) was employed on a neaSNOM (neaspec/attocube systems AG, Germany) platform equipped with PtIr coated atomic force microscopy (AFM) Si probes (Warsash Scientific Pty, Australia) of 260 kHz nominal resonance frequency and contact radius better than 25 nm. Topography data was acquired employing AFM tapping mode and optical amplitudes and single point IR absorbance spectra were recorded using a broadband infrared laser (600–2100 cm^−1^) focused onto the metallised AFM tip. More details about the method can be found in Huth et al. [21]. Typical data acquisition parameters are 80 nm tapping amplitude, 10–20 ms integration times and at least three averages/point for each spectrum. Spectral data was processed (phase tilt and offset) utilising neaPLOT (ver. 1.9.719) software.

#### 2.2.5 Nanoindentation

Nanoindentation tests were performed using a Hysitron TI950 Triboindenter with a Berkovich indenter (three-sided pyramidal indenter with a tip radius of approximately 150 nm and included angle of 142.3⁰) at room temperature. Tip area function of the Berkovich indenter was carefully calibrated on fused quartz with known reduced modulus (69.6 GPa ±5 %) and hardness (9.25 Gpa ±10 %). Depth profile (variation of mechanical property with respect to indentation depth) was obtained by using a partial unloading test.

Reduced modulus *E*_*r*_ and hardness *H* were extracted from the load-displacement curve, according to the Oliver-Pharr method (Oliver & Pharr, 1992), as:

$$E_{r}=\frac{\sqrt{\pi }}{2}\frac{S}{\sqrt{A}}$$

$$H=\frac{P_{\max}}{A}$$

where, *S* is the measured stiffness, *P*_max_ is the peak load, A is the contact area. Reduced modulus *E*_*r*_ is a combined modulus of the sample and the indenter, which is given by:

$$\frac{1}{E_{r}}=\frac{\left(1-v^{2}\right)}{E}+\frac{\left(1-{v_{i}}^{2}\right)}{E_{i}}$$

where, *E* and *v* are the elastic modulus and Poisson's ratio of the sample, *E*_*i*_ and *v*_*i*_, are the elastic modulus and Poisson's ratio for the indenter. For a standard diamond indenter probe, *E*_*i*_ is 1140 Gpa and *v*_*i*_ is 0.07.

Quasi-static nanoindentation tests with 200 nm peak displacement were also performed for these samples, with 5 s loading, 5 s holding and 5 s unloading.

#### 2.2.6 Nanoscratch test

Nanoscratch tests were performed using the Hysitron TI950 Triboindenter with a 1 μm tip radius cono-spherical indenter at room temperature. A 6 μm constant load scratch under 500 μN with a 10 μm pre-scratch line scan and 10 μm post-scratch line scan was performed on sample surface. The 10 μm pre-scratch line scan was used to correct the tilt of the surface, while the 10 μm post-scratch line scan was used to measure the residue scratch impression. The measured normal displacement *vs.* lateral displacement under scratching for different samples are shown in Fig. S2. An *in-situ* SPM imaging scan (20 μm × 20 μm) was also performed after the nanoscratch test to examine the scratch impression, as shown in Fig. S2.

#### 2.2.7 Viscoelasticity mapping by AMFM

All experiments have been done using a Cypher S AFM (Asylum Research/Oxford Instruments, Santa Barbara, CA) in AM-FM (Amplitude Modulation-Frequency Modulation) viscoelasticity mapping mode employing high aspect ratio ETALON HA-NC (TipsNano, Estonia) probes with nominal resonance frequency *f*_N_ = 140 kHz and nominal spring constant *k*_N_ = 3.5 N/m. All probes have been mounted in a high frequency/low noise AMFM cantilever holder. For the AM pass probes were actuated to free air amplitude of 2.0 V at the nominal resonant frequency and engaged at 800 mV set point. The second pass was done actuating for the harmonic at approximately 790 kHz for free air amplitude of 25 mV, while maintaining phase to 90°. Sample engagement was done so that probe tapping was in repulsive mode at a phase around 40°for the AM pass. Before sample analysis, the cantilever's conversion constant was determined fitting the Young's modulus (E = 1.2 GPa) of a known sample (UHMWPE-8456 standard reference material from National Institute of Standards and Technology, USA) at 22 ± 0.2 °C. A typical experiment involves the simultaneous acquisition of topography, frequency, dissipation, indentation and the calculation of stiffness, elasticity and loss tangent. More details about the method are described in Refs. [22,23].

#### *2.2.8* *In vitro* release of TA

Scaffolds coated with 1%HSA/10%TA and 5%HSA/1%TA were placed in low protein binding tubes (Eppendorf, Germany) and immersed in 1 mL of phosphate-buffered saline at pH 7.4. Samples were incubated at 37 °C, under 100 rpm for 7 days. Supernatants were collected at 1h, 3h, 6h, 12h and 1, 3 and 7 days and stored at −20 °C, until used for subsequent HPLC analysis.

#### 2.2.9 High performance liquid chromatography (HPLC) with UV detector

TA was first extracted from the *in vitro* release supernatants, as they contained HSA that could affect the HPLC column and TA detection, by following a modified protocol described by Cass and Bur [24]. Briefly, 500 μL of each sample were added to 500 μL of 1 M sulfuric acid, samples were then vortexed and 2 mL of ethyl acetate were pipetted into the vials. After overnight shaking, samples were centrifuged at 1800×*g* for 15 min and the organic phase was pipetted into new glass vials. Solutions were allowed to evaporate overnight and the next day, the residues were re suspended in 500 μL of methanol and transferred to HPLC vials for analysis. Extracted samples in addition to a standard curve prepared with known concentrations of TA, were analyzed using a HPLC 110 Series System with Autosampler and Diode Array Detector (Agilent Technologies, USA) with a reverse phase C18 column (GL Sciences, Japan). The mobile phase used consisted of 50 % methanol in water with 0.5 % phosphoric acid. A volume of 50 μL of each sample was injected into the system for analysis with a flow rate of 1 mL/min and a detection wavelength of 254 nm.

#### 2.2.10 Quartz crystal microbalance (QCM)

Immobilization of HSA and TA on mPCL spin-coated QCM sensors was quantified using a Q-sense system (Biolin Scientific, Sweden). Briefly, 14 mm Cr/Au QCM sensors with a fundamental frequency of 5 MHz were spin-coated with a 2 % mPCL w/v solution in chloroform for 30 s at 3500 rpm. Coated sensors were then plasma-treated for 4 min at medium power and subsequently used for either 1%HSA/10%TA and 5%HSA/10%TA treatment. Coated sensors were placed in the QCM chamber and the change in frequency (ΔF) and dissipation (ΔD) with the 3rd, 5th and 7th harmonics were recorded. Initially, buffer solution was pumped into the chamber at a flow rate of 50 μL/min until a stable baseline was achieved. Once the signal was stabilized, HSA was passed into the system for 5 h, then PBS was pumpled for 10 min in order to rinse the sensor surface. Finally, TA solution was injected to allow binding. Collected data were analyzed using the software QSense Dfind, and the mass density of immobilized HSA and TA was quantified using viscoelastic modelling.

#### 2.2.11 Contact angle measurement

Contact angle of unmodified and modified scaffolds was measured using Biolin ThetaFlex drop shape. Briefly, a drop of water (3 μL) was deposited on the surface of flat scaffolds and the contact angle was measured.

#### 2.2.12 3D broth assay

Treated and untreated scaffolds were incubated in 0.5 mL of *S. aureus* (ATCC 29213) suspension at a concentration of 1 × 10^8^ CFU/mL at 37 °C for 3 days. At each time point (day 1, 2 and 3), scaffolds were separated for analysis and the rest was used to continue the experiment until day 3. In the case of analysis, scaffolds with adherent bacteria were transferred to another well plate, washed three times with PBS in order to remove unadhered bacteria and used for CFU counting and SEM imaging. Remaining scaffolds used to continue the experiment, were placed in another well plate and fresh LB media was added at each time point.

#### 2.2.13 CFU counting

Scaffolds were placed in 1.5 mL low-binding Eppendorf tubes with 1 mL PBS. Adhered bacteria to the scaffolds surface were detached by two 15 min-cycles of sonication at 100 rpm, with a 10-s vortexing step, prior and post sonication. Resulting bacteria suspension was serially diluted in PBS and 5 μL aliquots of each suspension were plated onto LB agar and incubated overnight at 37 °C. After incubation, CFU were counted.

#### 2.2.14 SEM imaging

Scaffolds were fixed for 3h with 2.5 % glutaraldehyde, and then washed three times with PBS. The samples were then dehydrated via a series of ethanol treatment with increasing concentrations from 10 % to 100 % (%v/v) by using a Pelco BioWave Microwave Tissue processor. Once dehydrated, the scaffolds were coated with a 5 nm layer of platinum using a Leica EM-SCD005 cool sputter coater 7001F (Leica Microsystems GmbH, Germany). Adhered bacteria to the coated and uncoated scaffolds were then imaged using SEM (Tescan MIRA3 FEG-SEM, Australia) at a voltage of 3 kV (spot size 11.86).

#### 2.2.15 Bioluminescence measurements

The bacteria strain *S*. *aureus* Xen 36 possessing a stable copy of the *Photorhabdus luminescens luxABCDE* operon on the native plasmid, was purchased from PerkinElmer (USA). Bacteria were maintained in LB medium at 37 °C under ambient aeration as recommended by the supplier. Control and HSA/TA-coated scaffolds were incubated with 1 × 10^8^ CFU/mL *S. aureus* Xen 36 overnight, scaffolds were then transferred to a new well, washed twice to remove unattached bacteria, and incubated with 500 μL XenoLight D-Luciferin Potassium Salt for bioluminescence imaging using a Xenogen IVIS Spectrum (PerkinElmer, USA).

#### 2.2.16 Transmission electron microscopy (TEM)

Treated and untreated scaffolds were incubated in 0.5 mL of *S. aureus* (ATCC 29213) suspension at a concentration of 1 × 10^8^ CFU/mL at 37 °C. After 24h, the scaffolds were transferred to another well plate and washed twice with 0.1M sodium cacodylate buffer in order to remove non-adhered bacteria. Later, a pre-fix step was performed by incubating the samples in wash buffer (0.1 M sodium cacodylate with 2 % ruthenium red) for 5 min, the pre-fix solution was then replaced with the fixation solution composed of 2.5 % glutaraldehyde in 0.1 M sodium cacodylate with 2 % ruthenium red. After 2 h of fixation and staining, the samples were rinsed with wash buffer three times for 5 min, and posteriorly washed with only 0.1 M sodium cacodylate buffer in order to remove the ruthenium red excess from the samples. Scaffolds were then incubated in 1 % osmium tetroxide in 0.1 M sodium cacodylate buffer to cross-link lipids and embed a heavy metal in the cell membranes. For this, a Pelco BioWave Microwave Tissue processor was used with two cycles of 2 min vacuum with 2 min break in between both cycles. Samples were then rinsed with 0.1 M sodium cacodylate buffer three times for 5 min. Dehydration through graded ethanol series of 20 %–100 % were performed using the Pelco BioWave Microwave Tissue processor. After dehydration, samples were embedded using Epofix (Struers, Denmark), a cold curing, prepolymerised homopolymer resin kit supplemented with a hardener, through graded resin in ethanol ratios, 1:2, 2:1 and 100 %. Once embedded, samples were sectioned using an ultramicrotome (Leica, Germany) and posteriorly imaged using a 1400 TEM (JEOL, Japan).

#### *2.2.17* *In vitro* biocompatibility study

Human preosteoblasts (hOB) were isolated by explant culture from a male patient undergoing hip arthroplasty following written informed consent (approved by QUT Human Research Ethics Committee approval number 1400001024). Primary hOB cell sheets were formed following previously established protocols [[25], [26]]. Briefly, cells were seeded at a density of 20.000 cells/cm^2^ in 12-well-plates and cultured with alpha MEM supplemented with 10 % fetal bovine serum (FBS) and 1 % penicillin/streptomycin (p/s). Once cell layers were confluent, the culture media was further supplemented with osteogenic factors (10 mM β-glycerophosphate, 0.17 mM ascorbate-2-phosphate and 100 nM dexamethasone) in order to promote cell differentiation and matrix deposition. After 2–3 weeks, cell sheets were cultured for 7 more days with media supplemented with 0.17 mM ascorbate-2-phosphate only to achieve mechanically stable cell sheets that can be easily peeled off the wells as one entity. Cell sheet constructs were detached from each well and wrapped around control and HSA/TA-coated scaffolds and further cultured for 14 days in medium with no osteogenic factors, cell sheets were then collected for confocal and scanning electron microscopy imaging.

#### 2.2.18 Statistical analysis

A minimum of four experimental replicates (unless otherwise mentioned) were used in each study and the results are presented as mean value ± standard deviation. The effect of HSA/TA surface treatment in each assay compared to the controls was analyzed using two-way ANOVA (GraphPad Prism 9 software, USA). Differences between the groups were analyzed using the Tukey test of multiple comparisons and a confidence level of *p* < 0.05) was considered as statistically significant, unless otherwise specified.

## 3 Results and discussion

### 3.1 Structural insights and molecular dynamics into the binding mechanism of the HSA/TA complex

**Fig. 1:**
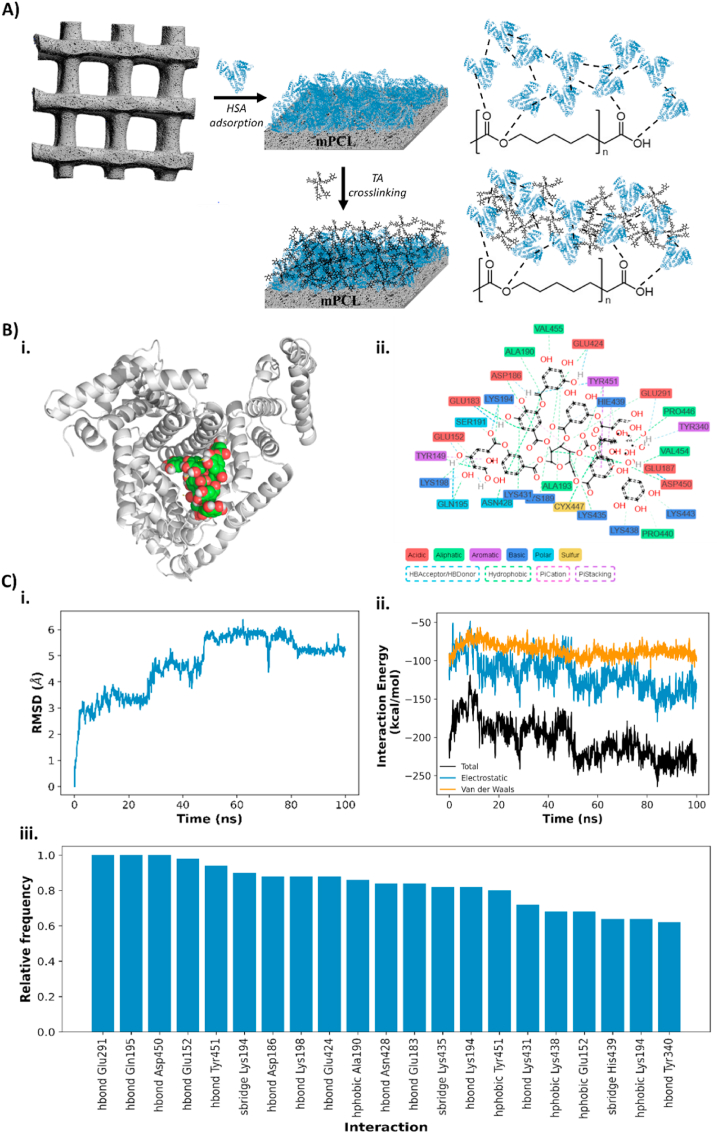
HSA/TA coating of microporous mPCL scaffolds. A) Schematic representation of HSA and TA immobilization on microporous mPCL scaffolds. B) Binding mechanism of the HSA/TA complex using molecular docking; i. Visual representation of the HSA/TA complex conformation with the highest docking score; ii. Non-covalent interactions and residues involved in the binding of TA to HSA; C) MD simulation analysis. i. RMSD vs time. ii. Electrostatic and van der Waals energy contributions to the binding and iii. Relative frequency of the interactions presented in the last 50 ns of the MD simulation.

Human serum albumin (HSA) is used in various FDA-approved medical devices and pharmaceutical applications, including its role as a cancer drug delivery vehicle via intravenous injection (e.g., Abraxane®). HSA is preferred due to its many advantages, including good biocompatibility, high drug encapsulation efficiency, low toxicity, good water solubility, and non-immunogenicity [27,28]. Conversely, tannic acid (TA) is a high molecular weight water-soluble polyphenol that has been widely used as a food additive due to its excellent antibacterial activity and free radical scavenging ability, making it a natural preservative in the food industry [29]. Tannic acid (TA) readily forms stable complexes with macromolecules via multiple binding sites through hydrogen bonding, hydrophobic and electrostatic interactions. Consequently, TA has been widely used as a crosslinker in biomaterials like collagen and hyaluronic acid, enhancing their thermal stability and mechanical properties [30,31]. Due to their pharmacological activities, the combination of HSA and TA has excellent potential for development and application as antibacterial coating for medical devices [15]. In our study, we utilized TA to stabilize a layer of HSA that was previously adsorbed onto the surface of 3D printed microporous mPCL scaffolds. The interaction between TA and HSA not only promotes the stabilization of HSA, but it also allows the immobilization of TA on the surface, resulting in a uniform HSA/TA coating evenly distributed across the mPCL scaffolds (Fig. 1A).

This study employed a molecular docking approach to investigate the binding mechanisms between HSA, a protein found in blood plasma, and TA, a polyphenolic compound. TA adopted nine different conformations while interacting with HSA, out of which six were found to be most favorable. These six conformations were observed to be located near the hydrophobic pockets in subdomains IIA and IIIA of HSA. It is worth noting that these pockets have been previously reported to have a high affinity for polyphenols, which are known to exhibit antioxidant and anti-inflammatory properties [32]. Herein, we use the conformation with the highest score as representative of the binding mode (Fig. 1Bi). As observed in Fig. 1Bii, there is a wide variety of non-covalent interactions and residue types involved in the binding of TA with HSA. Particularly, the main interactions calculated were hydrophobic, seconded by hydrogen bonds. This was expected due to the size of the TA ligand and the high number of hydroxyl groups in the structure and is consistent with previous reports studying the interactions of other polyphenols, including chlorogenic acid, silibinin and saikosaponin C, with HSA [[33], [34], [35]]. Despite the presence of several aromatic rings in the ligand, few aromatic residues are present and only two π-stacking interactions were observed.

To validate the docking simulation and evaluate the stability of the compound in the binding site along with the interactions involved in the molecular recognition, a 100-ns molecular dynamics simulation was performed. Fig. 1Ci shows the root-mean-square deviation of atomic positions (RMSD) of the TA structure throughout the simulation. The RMSD stabilizes after 50 ns, suggesting that the structure has adopted the most stable conformation. After stabilization at 50 ns, electrostatic contribution to the interaction energy drops in comparison to the van der Waals interactions (Fig. 1Cii), indicating that binding is driven mainly by hydrogen bonding and ionic interactions. Fig. 1Ciii presents the interaction between TA and HSA with a relative frequency greater than 0.5. Based on these results, the binding mechanisms appear to rely primarily on hydrogen bonding with the residues Glu291, Gln195 and Asp450 which are present in all the frames. Other important residues involved in the binding include Lys194, which forms three types of interactions: hydrogen bonding, salt bridges and hydrophobic; and Glu152 involved in hydrogen bonding and hydrophobic interactions. Insights into the intramolecular interactions between HSA and TA are essential for the future optimization of this system in order to improve binding and stability. Further studies could explore longer MD simulations and other solvent systems to offer a better understanding of TA and HSA complex stability.

### 3.2 Surface topography, chemical composition and mechanical properties of HSA/TA-coated scaffolds

**Fig. 2:**
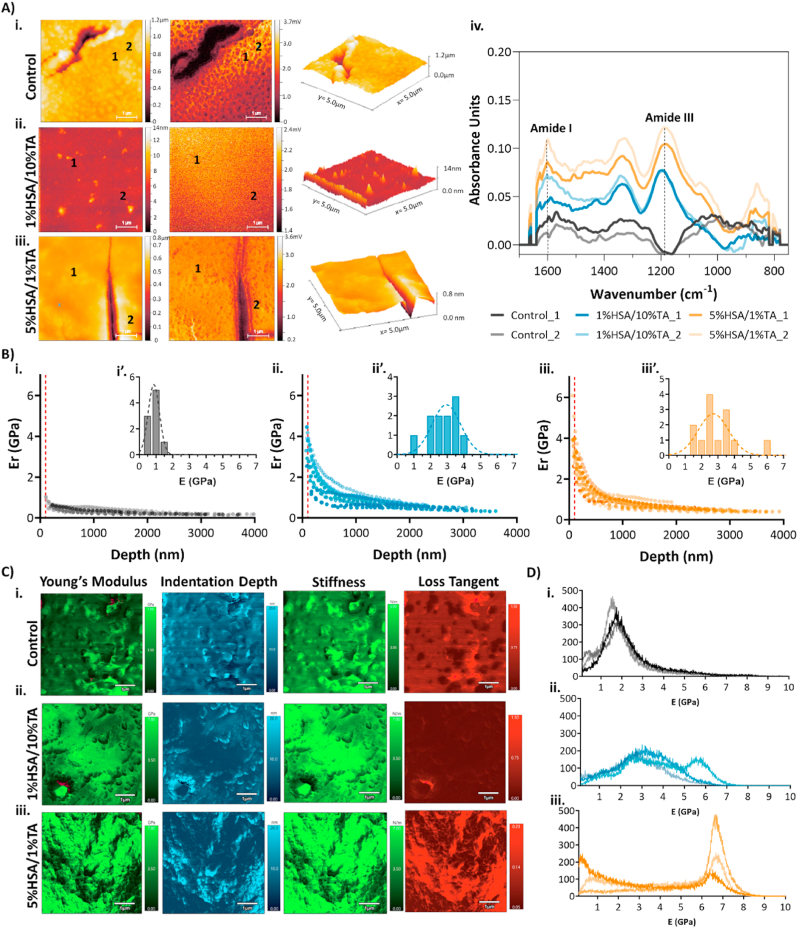
Surface topography, chemical composition, and mechanical properties of HSA/TA-coated scaffolds**.** A) surface height profiles, 3D reconstruction and IR optical amplitude of unmodified and HSA/TA-treated surfaces by nanoFTIR. IR spectra were acquired at two representative areas of each scaffolds surface, the location of both areas is indicated with the numbers 1 and 2 on the AFM height and nanoFTIR reflectivity panels. Mechanical characterization of unmodified and HSA/TA-coated scaffolds by nanoindentation (B) and AFM nanomechanical mapping (C). B) Depth profiles of i. control, ii. 1%HSA/10%TA and iii. 5%HSA/1%TA scaffolds; insets (i’-iii’) showing the elastic modulus distribution of the respective surfaces, calculated at 100 nm indentation depth (red dashed line in depth profiles). C) AFM nanomechanical mapping of i. control, ii. 1%HSA/10%TA- and iii. 5%HSA/1%TA-coated surfaces. D) Young's moduli histograms for i. control ii. 1%HSA/10%TA and iii. 5%HSA/1%TA scaffolds. Histograms include the measurement of three different scaffolds. Scale bars: 1 μm.

In order to characterize the topography and chemical composition of both 1%HSA crosslinked with 10%TA and 5%HSA stabilized with 1%TA coatings at a nanoscale resolution, nanoFTIR, a method that allows infrared spectral acquisition from topographical features permitting nanoscale chemical identification and hyperspectral imaging was used. Fig. 2A shows the topographical features present on the surface of the control, 1%HSA/10%TA- and 5%HSA/1%TA-coated scaffolds, as well as the IR spectra collected from two representative areas on the surface. As observed in Fig. 2Ai-iii, HSA/TA-modified scaffolds had significantly different surface morphologies in comparison to the porous control scaffolds, as a result of HSA and TA immobilization. Both 1%HSA/10%TA- and 5%HSA/1%TA-coated scaffolds showed a smooth and regular surface with the presence of a few nanoscale peaks in the case of 1%HSA/10%TA.

The IR spectra from two representative locations of each surface were acquired. As observed in Fig. 2, Fig. 1%HSA/10%TA- and 5%HSA/1%TA-coated scaffolds showed a common peak at 1250-1350 cm^−1^ corresponding to the band of amide III, which derives mainly from the bending vibrations of amide N-H, C-N and minor contribution from C=O bending [36]. Amide I bands (1600-1700 cm^−1^) were also identified on HSA/TA-modified surfaces, however, these peaks overlap strongly with the characteristic C=O peak of PCL (1740-1750 cm^−1^). Interestingly, both scanned areas for all the surfaces revealed similar surface chemistry, confirming the homogeneous distribution of HSA on both coated surfaces at the nanoscale.

In addition to studying the topographical and chemical composition of the HSA/TA-modified surfaces, it is important to determine the mechanical properties of these coatings as they should possess adequate chemical and mechanical stability and adhesion to the biomaterial surface to resist surgical insertion and repeated mechanical loading during implantation. Herein, we use nanoindentation, AFM nanomechanical mapping and nano-scratch testing to characterize the mechanical stability of HSA/TA-coated scaffolds.

First, the mechanical response of unmodified and coated scaffolds at different indentation depths was obtained by using a nanoindentation partial unloading test. Fig. 2Bi-iii shows the change in the reduced elastic modulus (*Er*) at indentations depths ranging from 0.1 to 4 μm. In all the cases, *Er* values were higher at low penetration depths, and decreased as the indenter penetrated further into the samples, until it reached a plateau at around 1 μm. The distinctive *Er* profiles at different indentation depths through the coatings suggest a significant contribution from the substrate's mechanical properties (mPCL). This is because the elastic field under the indenter is not confined to the coating itself, but it is rather a long-range field that extends into the substrate, especially when the coating thickness is small (Fig. S1). As a result, the indentation response of a thin film (coating) on a substrate is a complex function of the elastic and plastic properties of both the film and substrate. A widely accepted alternative to measure ‘film-only’ properties is to restrict the indentation depth to less than 10 % of the film thickness, however, this can be difficult due to experimental constrains such as surface roughness and substrate inhomogeneities [37,38]. It was demonstrated previously that 1%HSA/10%TA- and 5%HSA/1%TA-coated scaffolds have thicknesses of *ca.* 2.2 and 3.8 μm, respectively [15]. Therefore, we extrapolated the elastic modulus from the respective depth profiles at 0.1 μm (<10 % of their total thickness), to limit the contributions from the substrate. Both coatings had superior mechanical properties at the micro-scale, with elastic moduli ranging from 1.2 to 4.5 GPa and 1.7–6.7 GPa in the case of 1%HSA/10%TA- and 5%HSA/1%TA-coated scaffolds, respectively, in comparison to 0.5–1.5 GPa in the case of control mPCL scaffolds (Fig. 2Bi’-iii’). Nevertheless, it should be noted that even at this low indentation depth, the mPCL substrate might be contributing to the measured mechanical properties, particularly, the randomly distributed microporosity on and within the scaffold makes it difficult to know when the indentation is happening on the coating that is deposited above or in the proximity of a micropore. To elucidate this effect, loading curves at a maximum indentation depth of 200 nm were performed on HSA/TA-coated samples on microporous and nonporous mPCL surfaces (Fig. S2). As observed in the SEM images and loading curves, the presence of micropores led to significant variations in the mechanical response of coatings.

To eliminate any effects from the bulk material properties, we conducted AFM nanomechanical mapping at indentation depths ranging from 0 to 20 nm. We then measured the Young's moduli and loss tangent of the surface, which are unique indicators of its viscoelastic properties, in a 5 μm × 5 μm surface area (Fig. 2C). Fig. 2Ci-iii shows the indentation maps including the Young's modulus, indentation depth, stiffness, and loss tangent throughout the scanned area. Histograms of the Young's moduli distribution on the unmodified and HSA/TA-treated surfaces evidenced superior mechanical properties when HSA and TA were present on the surface. The Young's moduli ranged from 4 to 8 GPa, for both 1%HSA/10%TA- and 5%HSA/1%TA-coated scaffolds, in comparison to a range of 0–4 GPa in the case of control mPCL scaffolds (Fig. 2D). Similarly, both HSA/TA-modified surfaces exhibited a lower loss tangent in comparison to the unmodified surfaces, suggesting that coated scaffolds have a superior elastic response as they present a lower potential for energy dissipation. It is worthwhile mentioning that there were not significant mechanical heterogeneities within each analyzed surface, suggesting that both coatings possess uniform properties at the nanoscale. Several studies have shown that the use of TA in hydrogel systems lead to enhanced mechanical properties as TA is able to crosslink polymer networks via hydrogen bonding [30,31,39]. Therefore, superior mechanical properties of HSA/TA-coated scaffolds in the comparison to the unmodified mPCL surfaces are a result of the stable complex formed between these two molecules.

To further characterize the mechanical stability of the coatings, nanoscratch tests at a constant load were performed. Fig. S3 shows the measured normal displacement vs lateral displacement of scratched control and HSA/TA-coated surfaces at three different stages: pre-scan, in which the surface is scanned; scratch, during which the indenter scratches along the surface; and residue, in which the surface is scanned after scratching. Interestingly, control scaffolds had the highest plastic deformation, seen as a deeper indentation that led to material pile-up on the groove sides and a significantly higher residue (Fig. S3A). In contrast, both coated scaffolds showed higher elastic recovery and an improved scratch resistance seen as a significantly lower residue in comparison to control surfaces (Figs. S3B–C). In comparison to uncoated mPCL scaffolds, those coated with HSA/TA demonstrated superior mechanical properties, which are crucial for their successful application as antibacterial implant coatings. Besides the characterization of the surface chemistry and mechanical stability, changes in surface wettability as a result of HSA and TA immobilization were also studied (Fig. S4). Control surfaces showed a water contact angle (WCA) of 73.9 ± 2.4°, while 1%HSA/10%TA- and 5%HSA/1%TA-coated scaffolds had significantly lower WCAs of 49.7 ± 4.2° and 52.4 ± 2.4°, respectively. The significant increase in surface wettability is attributed to the hydrophilic nature of HSA and TA.

### 3.3 Quantification of immobilized HSA and TA

The mass of HSA and TA immobilized on the mPCL surfaces was quantified by quartz crystal microbalance with dissipation monitoring (QCM-D) and high-performance liquid chromatography (HPLC). In the case of QCM-D, HSA and TA were immobilized onto gold QCM sensors spin-coated with mPCL to simulate the scaffold surface. The change in frequency (Δ*F*) and dissipation (Δ*D*) were simultaneously measured to obtain information about the mass change and the viscoelastic properties of the HSA/TA layers deposited on the sensor surface. An increase of mass on the sensor surface will be detected as a decrease in frequency, while a loss of mass will lead to an increase in the frequency reading. In comparison, the change in dissipation depends on the viscoelastic properties of the adlayers; a decrease in dissipation suggests stiffening of the deposited layer, whereas an increase in dissipation is correlated with a softer structure [40].

**Fig. 3:**
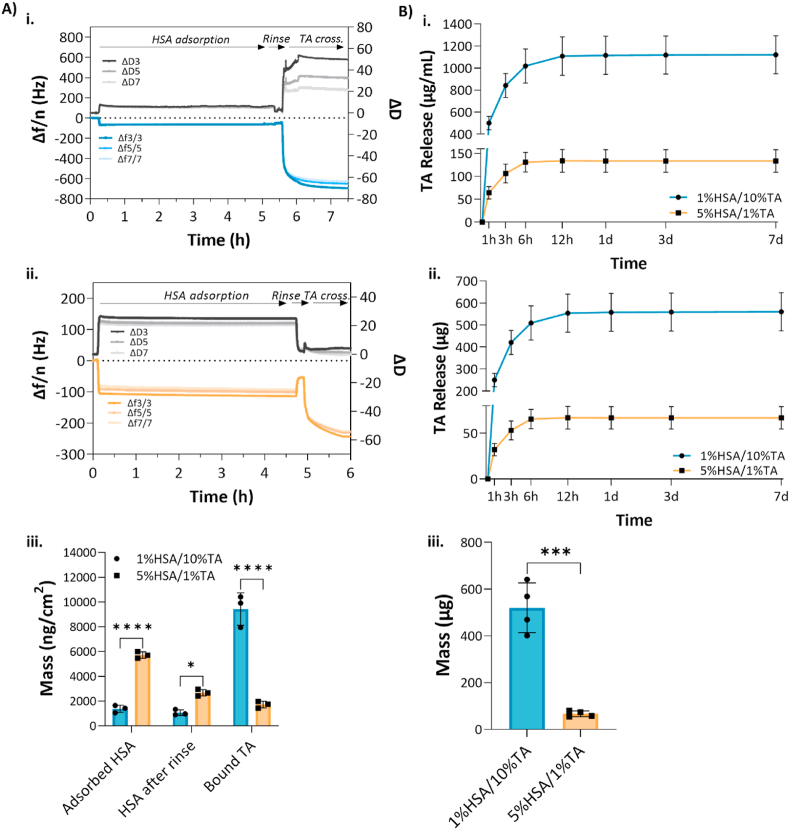
Quantification of immobilized HSA and TA on the mPCL surfaces A) QCM data showing the change of frequency (Δ*F*) and dissipation (Δ*D*) with the 3rd, 7th and 9th harmonics as a result of HSA adsorption and TA binding i. 1%HSA/10%TA and ii. 5%HSA/1%TA. iii. Quantification of HSA and TA deposition on mPCL spin coated QCM sensors using viscoelastic modelling. B) *In vitro* release of TA in phosphate-buffered saline at pH 7.4, under 100 rpm agitation at 37 °C. i-iii. Cumulative release of TA from 1%HSA/10%TA- and 5%HSA/1%TA-coated scaffolds. All measurements are reported as average ± standard deviation (SD), *p < 0.05; **p < 0.01; ***p < 0.001; ****p < 0.0001, (n = 4).

Fig. 3A shows Δ*F* and Δ*D* data collected during the immobilization of HSA and TA on the mPCL-coated QCM sensors when 1%HSA/10%TA and 5%HSA/1%TA were used. For both groups, the variations in frequency and dissipation during HSA adsorption and subsequent TA binding provided evidence of the changes in mass at each step of the sensor surface coating (Fig. 3Ai,ii). For instance, an initial continuous decrease of frequency was observed during the HSA adsorption, showing that HSA deposits on the sensor surface. After 5 h, the reaction was stopped and rinsed with buffer for 10 min, leading to an increase in frequency as unbound HSA molecules were washed off from the surface. After rinsing with buffer, TA was injected into the system, leading to a decrease in frequency as TA bound to the HSA-deposited surface. The calculated density of HSA and TA immobilized on the surfaces is shown in Fig. 3Aiii. Particularly, when the sensor was exposed to 1%HSA, followed by 10 % TA stabilization, 1.06 ± 0.23 μg/cm^2^ and 9.42 ± 1.31 μg/cm^2^ of HSA and TA were immobilized on the surface, respectively. In contrast, when 5%HSA and 1%TA were used, the density of immobilized molecules was 2.67 ± 0.26 μg/cm^2^ and 1.71 ± 0.27 μg/cm^2^ in the case of HSA and TA, respectively.

Even though QCM is a powerful and precise technique to monitor real-time changes in the mass and viscoelastic properties of adlayers, there are limitations to the use of this system for the study of HSA/TA coatings. Particularly, the sensors could only be spin-coated with mPCL instead of the microporous mPCL, as the reference layer where HSA and TA are deposited, needs to have a smooth and uniform surface topography, which is not possible with the microporous mPCL composite as this is composed of mPCL and sugar particles. Once the sugar is leached from the surface, it will lead to the formation of micropores and inhomogeneities on the surface that will interfere with the resonance frequency of the sensor and affect the validity of the acquired data. In addition, the rinsing step between HSA adsorption and TA binding had to be introduced to avoid unwanted reaction between both molecules in the system before reaching the sensor surface, however, this step is not performed when coating the microporous mPCL scaffolds. These differences in the immobilization process of HSA and TA on the QCM sensors and the microporous mPCL scaffolds affect the translatability of acquired QCM data on microporous mPCL scaffolds, however, it serves as a good estimation for the quantification of HSA and TA immobilization.

In order to determine the amount of TA immobilized on the microporous mPCL scaffolds as well as its release over time, HPLC was used. Fig. 3B shows the cumulative release profiles of TA from 1%HSA/10%TA- and 5%HSA/1%TA-coated scaffolds over 7 days in phosphate-buffered saline at 37 °C. Both groups displayed a burst release over the first 6 h. As expected, a higher concentration of TA used for the coating led to more TA being immobilized and released. Overall, 1%HSA/10%TA- and 5%HSA/1%TA-coated scaffolds released 520.6 ± 106.1 μg and 66.84 ± 12.25 μg of TA within 1 day of incubation, respectively. Future studies will include the *in vitro* release of TA in a more physiologically relevant media including enzymes and plasma proteins that mimic better the *in vivo* microenvironment. In this context, we hypothesize that the presence of these biological factors might lead to a sustained release of TA, compared to PBS.

### 3.4 *In vitro S. aureus* adhesion on HSA/TA-coated scaffolds over time

**Fig. 4:**
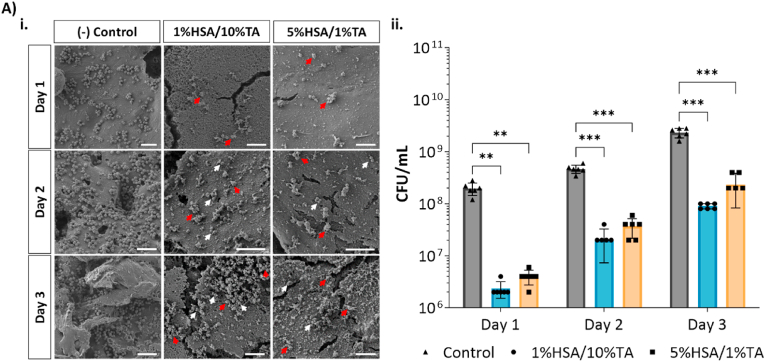
I*n vitro* evaluation of HSA/TA-modified scaffolds against *S. aureus* for 3 days. A) SEM images evidencing extensive bacterial colonization on the control surfaces in comparison to both 1%HSA/10%TA- and 5%HSA/1%TA -treated surfaces; Red arrows point at apparent dead bacteria while white arrows highlight viable bacteria cells B) Number of viable colony forming units of bacteria recovered from each scaffold at each time point. Data shown as mean ± SD, *p < 0.05; **p < 0.01; ***p < 0.001; ****p < 0.0001, (n = 9); scale bars: 5 μm.

The antibacterial efficacy of both 1%HSA/10%TA- and 5%HSA/1%TA-treated surfaces was assessed *in vitro* by exposing the scaffolds to *S. aureus* for 24 h and incubating them for three days in LB media to allow bacterial growth and biofilm formation. Bacterial colonization and biofilm formation on the unmodified and modified surfaces was followed by SEM imaging and CFU counting measurements over three days. Fig. 4A shows the SEM images of bacterial colonization on all the surfaces at each time point. After the first day, there was significant bacterial colonization on the control scaffolds. In contrast, 1%HSA/10%TA- and 5%HSA/1%TA-modified surfaces had a significant decrease in bacterial colonization of 98.8 ± 4.1 % and 98.0 ± 6.4 % in comparison to the control, respectively. After two and three days, control scaffolds demonstrated an increase in bacteria numbers as well as signs of biofilm formation. By comparison, even though there were signs of more viable bacterial cells (white arrows) adhering to HSA/TA-modified scaffolds during this time, there was still no evidence of biofilm formation and the significant difference in bacterial cell number between controls and modified surfaces was maintained (Table 1).

**Table 1:** Reduction in *S. aureus* colonization on HSA/TA-treated surfaces in comparison with the control surfaces.

Reduction in *S. aureus* colonization (%)	Day 1	Day 2	Day 3
1%HSA/10%TA - Control	98.8 ± 4.1	88.3 ± 6.8	46.3 ± 15.2
5%HSA/1%TA - Control	98.0 ± 6.4	79.1 ± 7.4	16.7 ± 23.8

Some studies have evaluated the antimicrobial properties of HSA and TA independently. For instance, An et *al.* showed that carbodiimide-crosslinked albumin-coated titanium implants had a significantly lower infection rate (27 %) in comparison to uncoated implants (62 %), on prosthetic infection in 29 adult male rabbits [41]. By comparison, Liu et *al*. demonstrated that biomedical catheters coated with hydrophobic TA were able to significantly decrease *S. aureus* colonization *in vitro* and *in vivo* without causing cytotoxicity nor inflammatory response [42]. In this study, we demonstrate for the first time, that 1%HSA/10%TA- and 5%HSA/1%TA-treated scaffolds are able to maintain their antimicrobial properties against *S. aureus* for 3 days, leading to a significant decrease in *S. aureus* colonization in comparison to the control microporous mPCL scaffolds.

One of the main limitations in assessing the antibacterial properties of biomaterials is the lack of noninvasive quantitative imaging techniques to monitor bacterial growth and biofilm formation. This limitation can be exacerbated by the biomaterial type and scaffold geometry. For instance, HSA and TA are highly reactive molecules that can interact with fluorophores and their target molecules such as DNA [43,44], rendering live imaging using fluorescence microscopy unreliable. Additionally, the 3D geometry of the scaffolds hinders imaging throughout the surface due to optical and technical limitations. The use of Luciferase-tagged bacteria represents a noninvasive and quantitative alternative to monitor bacterial proliferation on biomaterial surfaces *in vitro* and *in vivo*. As proof of principle, we used *S. aureus* Xen 36 to further characterize the antibacterial properties of HSA/TA-coated scaffolds. Fig. S5A shows the significant reduction of bacterial attachment on coated surfaces shown as negligible bioluminescence signals in comparison to control scaffolds. Interestingly, the bioluminescence signal in the bacteria suspension where the coated scaffolds were incubated was significantly lower in comparison to the unmodified surfaces, further demonstrating the antibacterial effect of components released from the coating over one day in culture (Fig. S5B). It is worth noting that although bioluminescent bacteria can help track infections over time without requiring the sacrifice of samples *in vitro* and animals *in vivo*, the detection limit is quite low at 10^5^ CFU [45]. This means that only a relatively high bacterial load can be detected, which is sufficient to cause a biofilm-related implant infection.

### 3.5 Antibacterial mechanism of HSA/TA coating

The exact antibacterial mechanism of HSA as well as TA remains unknown. Studies suggest that HSA can attach itself to bacterial cells and alter their surface properties. However, the molecular mechanisms and the specific properties that are affected are yet to be fully understood [46]. Other studies suggest that albumin binds and sequesters quorum sensing signaling molecules secreted by *P. aeruginosa*, interfering with the quorum sensing process in which bacteria moderate several vital processes [45]. In contrast, the antimicrobial properties of TA have been attributed to its ability to form complexes with iron cations that are essential for different vital metabolic processes [47], as well as with components on the outer cell membrane that can lead to membrane permeability, increased osmotic pressure and lysis [48].

SEM imaging of *S. aureus* upon contact with 1%HSA/10%TA- and 5%HSA/1%TA-coated surfaces (Fig. 4Ai) evidenced morphological changes on the outer cell wall of bacteria, suggesting possible membrane disruption as a result of the antimicrobial treatment. In order to further investigate these findings and elucidate the antimicrobial mechanism of HSA/TA coatings, AFM and TEM imaging were used to detect morphological alterations on the outer cell wall with a nanoscale resolution. In addition, AFM nanoindentation is used to assess the changes on the mechanical properties of bacteria once in contact with the HSA/TA coatings. Finally, molecular docking is used to compare the interaction of TA with known *S. aureus* drug targets. The results from this analysis aim to offer a complementary view to the *in vitro* adhesion assays previously reported in this study, while providing a better understanding of the coating antimicrobial mechanism.

**Fig. 5:**
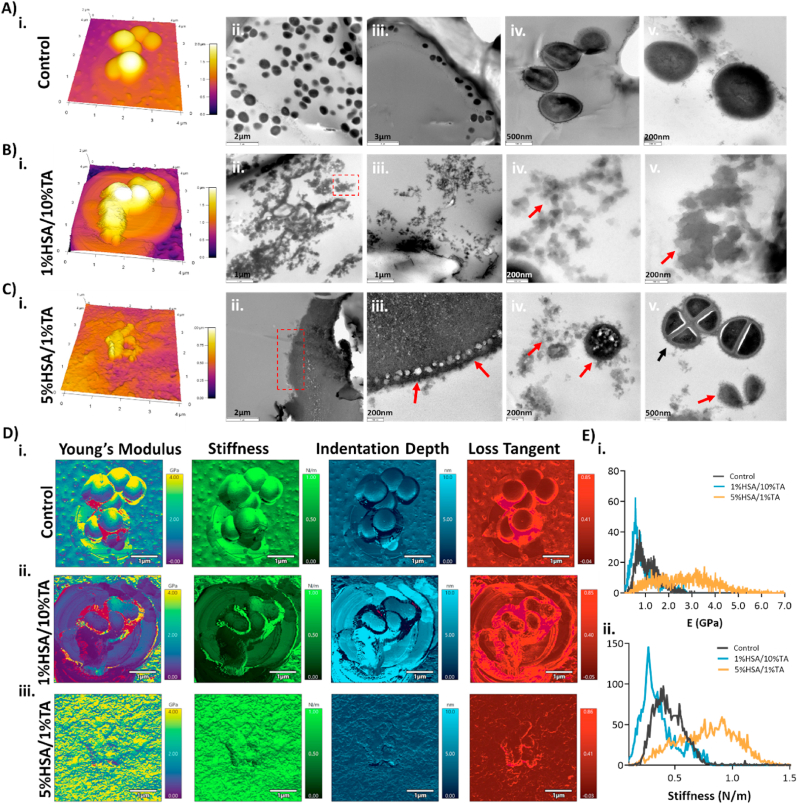
Morphological and mechanical changes on *S. aureus* outer membrane as a result of HSA/TA-treatment. AFM and TEM images of *S. aureus* present on A) Control B) 1%HSA/10%TA and C) 5%HSA/1%TA microporous mPCL scaffolds. Cells present on control scaffolds show normal morphology while bacteria exposed to the HSA and TA coating evidenced morphological changes suggesting loss of membrane integrity (red arrows). D) AFM nanoindentation on *S. aureus* bacteria in contact with) i. unmodified control substrates and ii. 1%HSA/10%TA- and iii. 5%HSA/1%TA-coated surfaces. E) Histograms presenting the distribution of i. Young's modulus and ii. Stiffness of *S. aureus*.

Fig. 5A–Ci, shows the surface morphology of bacteria present on the untreated and HSA/TA-modified scaffolds obtained with AFM. Bacteria present on the control surfaces showed typical spherical-shaped, smooth, and uniform structures, while bacteria present on HSA/TA surfaces showed rather different morphologies with rougher surface textures. Rougher morphologies of bacteria outer cell membrane have previously been reported as a sign of deformation because of antimicrobial treatment with other agents such as melittin, chitosan and polyelectrolytes [[49], [50], [51]]. Notably, bacteria exposed to HSA/TA coatings showed flatten morphologies with fusing effects with the surface. In addition, a possible layer of additional materials could have deposited on the surface of bacteria, this effect is more evident on 5%HSA/1%TA surfaces (Fig. 5Ci). Eaton et *al.*, described similar effects when exposing *S. aureus* and *E. coli* to chitosan, the authors suggested that this polymeric layer could be composed of intracellular material as a result of membrane leakage or extracellular polymeric substance [50].

To further investigate the structural changes observed with AFM as well as to detect possible intracellular alterations, TEM was used. Bacterial outer membranes of untreated cells exhibited smooth and uniform healthy structures (Fig. 5Aii-v), while we could not identify any normal structure corresponding to bacterial cells for the 1%HSA/10%TA group (Fig. 5Bii-v), suggesting a high degree of cell disruption (red arrows). In comparison, bacteria exposed to 5%HSA/1%TA (Fig. 5Cii-v) were found enclosed in a matrix, with some of the bacteria presenting clear membrane damage (red arrows) (Fig. 5Civ) and others undergoing division in seemingly good conditions (black arrows) (Fig. 5Cv). These findings agree with the AFM images in which bacteria appear to be covered in other components. We hypothesize that the coating could be interacting strongly with bacteria thereby isolating and inhibiting adsorption of nutrients as well as cell communication, which could lead to cell death.

Additionally, AFM viscoelasticity mapping was performed in order to obtain information about the mechanical properties of bacteria that could help explain the morphological changes observed with AFM and TEM. Bacteria present on the unmodified control substrates exhibited a relatively heterogeneous elasticity map, evidenced by a large Gaussian distribution. In comparison, bacteria exposed to 1%HSA/10%HSA surfaces appeared to have a softer structure than the other groups, with a narrow Gaussian distribution and a lower elastic modulus and stiffness range. Interestingly, bacteria exposed to 5%HSA/1%TA had a more heterogeneous elasticity map, suggesting stiffer structures (Fig. 5Ei-ii), nevertheless, the range and Gaussian distribution is very similar to the substrate (Fig.S6Bii-iii), suggesting that the material deposited on the surface of bacteria is also present on the surface of the scaffolds. Therefore, it could be possible that the coating is interacting and depositing on bacteria. It is important to note, that the mechanical properties reported in this study do not match the elastic modulus of *S. aureus* reported in the literature [50,52,53], as these reports imaged viable bacteria, while our study investigated the mechanical properties of fixed and dehydrated bacteria, due to sample preparation requirements for this technique.

The results obtained from the morphological and mechanical characterization of bacteria exposed to the HSA/TA coatings strongly suggest that the coating causes irreversible damage to the bacterial outer membrane, which is seen as softer and ruptured structures, as well as a deposited layer of materials on bacterial cells, which could originate from inner cellular contents or coating components.

To further understand at a molecular level the interactions between the coating and the outer cell membrane of *S. aureus*, a molecular docking study was performed. The possible interactions of TA with different known *S. aureus* target molecules were identified and later compared with different known inhibitors found in the literature.

Five PDB (protein data bank) structures containing known *S. aureus* drug targets in complex with an inhibitor were used to calculate the non-covalent interactions involved in the binding mode of each. Later, the estimation of free energy of binding and non-covalent interactions of TA with those drug targets were calculated by molecular docking. The five chosen *S. aureus* targets for this study were DNA gyrase subunit B (GyrB), dihydrofolate reductase (DHFR), thymidylate kinase (TMK), enoyl-[acyl-carrier-protein] reductase (FabI*)* and the Accessory gene regulator (Agr), all of which play key roles in DNA and fatty acid synthesis as well as cell communication and division [[54], [55], [56], [57], [58]].

GyrB is a well-known drug target used in the development of antibacterial agents as it plays a key role in the bacterial gene expression mediated by DNA supercoiling [54]. Several compounds have been proposed as inhibitors of GyrB, such as coumarin, pyrroles, pyrazolopyridones and pyrrolamides [59]. The crystal structure of *S. aurerus* Gyrb in complex with kibdelomycin, a natural compound isolated from *Kibdelosporangium* sp., was used to identify the key residue involved in the inhibition of this enzyme. This ligand makes several hydrophobic contacts which mediate the binding. Some hydrogen bonds with Glu58, Gln91, Lys93, Gly109 and Gly110 are observed. The docking score of kibdelomycin and GyrB was −9.7 kcal/mol. In contrast, TA interacts with more residues presumably due to its larger size. Comparison of these interaction profiles is indicated in Fig. S7A. It can be seen that TA forms most of the interactions observed with kibdelomycin, interacting mainly with Glu58, Gln91, Gly109 and Thr173 by hydrogen bonds. However, the docking score for TA was also −9.7 kcal/mol, which indicates that newer interactions did not contribute greatly to affinity. In addition to the crystalized ligand, other known inhibitors were docked to compare their binding mode and to identify key interactions. Clorobicin and novobiocin, both coumarins derivatives, exhibited docking scores of −8.5 kcal/mol and −7.5 kcal/mol, respectively, showing similar interactions, including a hydrogen bond with Arg84 which is also shared with TA but is not present in kibdelomycin's profile. These findings suggest that TA might have a potential effect on GyrB, analogous to that of the other inhibitors studied.

DHFR, is crucial for maintaining intracellular levels of 5,6,7,8-tetrahydrofolate and its byproducts, which are necessary cofactors in the production of several metabolites. This enzyme's inhibition prevents DNA synthesis and cell division, which results in cell death [56]. Iclaprim is a diaminopyrimidine inhibitor of DHFR in methicillin-resistant *S. aureus*, which has demonstrated robust and quick bactericidal effects against the main Gram-positive infections. This compound interacts with DHFR mainly by hydrogen bonding with Leu5, Val6, Ala7, Leu20, Ser49 and Thr111. Nevertheless, literature indicates that hydrophobic interactions contribute to affinity more than hydrogen bonds [60]. Therefore, interactions with Gln19, Leu20, Leu28, Val31, Ile50, Leu54 and Phe92 are more important. These interactions are conserved in profiles of the diaminoquinazolines M23, Q12, Q19, Q20, Q26 and Q27 (Li et al., 2011), as well as in TA (Fig. S7B). Notably, TA had a docking score of −10.8 kcal/mol, which was higher than best of the known inhibitors iclaprim (−9.2 kcal/mol). This could be related to those hydrophobic interactions formed in the complex with TA.

TMK is an enzyme that catalyzes the phosphorylation of thymidine 5′-monophosphate to thymidine 5′-diphosphate, an essential step in DNA synthesis [55]. Therefore, it is an attractive antibacterial drug target. Piperidinylthymine derivatives are the main inhibitors of this enzyme, which interact by hydrogen bond with Arg70 and Gln101 along with π-stacking with Phe66. Also, a salt bridge is formed with Arg48 and there are hydrophobic interactions with Arg48, Leu52, Arg92 and Tyr100 [61]. Based on interaction profile, TA forms most of these interactions and some other several hydrogen bond and salt bridges interactions (Fig. S9C). In addition, its docking score (−12.2 kcal/mol) was higher than the known inhibitors 14D (−10.3 kcal/mol), 32C (−9.8 kcal/mol), 31Z (−9.5 kcal/mol) and 32K (−9.2 kcal/mol). This indicates the potential binding of TA to TMK, which should be explored in more detailed studies.

Fabl is involved in the lipid and biotin biosynthesis by catalyzing the reduction of a carbon-carbon double bond in an enoyl moiety that is covalently linked to an acyl carrier protein (ACP) [57]. Structures of Fabl in complex with triclosan and hexachlorophene shows that halogen bonds play an important role in its inhibition along with the presence of π-stacking and hydrophobic interactions (Fig. S7D) [62]. Although TA generates several interactions, its structure does not contain a halogen atom, unlike triclosan. In addition, based on the docking score, TA (−7.1 kcal/mol) was worse than triclosan (−7.9 kcal/mol) and hexachlorophene (−8.3 kcal/mol). In this case, it is not clear the effect that TA could have on this enzyme.

Finally, the Agr system controls the expression of virulence factors and other genes in response to population density. The Agr system consists of two divergent transcriptional units, RNAII and RNAIII, and is regulated by the AgrA and AgrC proteins in response to auto-inducing peptides [58]. No crystal structure of the inhibitor bound to these targets has been established yet. However, some molecular docking studies have been conducted to investigate the interactions of various compounds with the Arg system. Savirin and thymol have been shown that hydrogen bonds with Arg218 and π-stacking with Tyr229 are the key interactions for its binding. TA also forms these interactions and exhibits a docking score of −5.8 kcal/mol (Fig. S7E). This score is more favorable than that of thymol (−4.7 kcal/mol), but slightly lower in comparison to savirin (−6.1 kcal/mol).

Overall, the results from the molecular docking study showed that TA has comparable or higher affinity with GyrB, DHFR, TMK and Agr, in comparison to known inhibitors (Table 2), which offers a preliminary view of possible target candidates that play a key role in TA antimicrobial mechanisms. Nevertheless, further studies using molecular dynamic simulations and experimental validation are necessary to establish the stability of these interactions, and their role in inhibiting bacterial growth and biofilm formation.

**Table 2:** Docking scores of TA and other inhibitors with known *S. aureus* targets.

*S. aureus* target	Inhibitor	Docking score (kcal/mol)
*DNA gyrase subunit B*	Tannic acid	−9.7
	kibdelomycin	−9.7
	Clorobiocin	−8.5
	Novobiocin	−7.5
*Dihydrofolate reductase*	Tannic acid	−10.8
	iclaprim	−9.2
	Q27	−8.9
	Q26	−8.8
	Q20	−8.1
	M23	−8.1
	Q12	−8.0
	Q19	−8.5
	Ar-102	−7.4
*Thymidylate Kinase*	Tannic acid	−12.2
	14D	−10.3
	32C	−9.8
	31Z	−9.5
	32K	−9.2
*Enoyl-[acyl-carrier-protein] reductase*	Hexachlorophene	−8.3
	Triclosan	−7.9
	Tannic acid	−7.1
*Accessory gene regulator*	Savirin	−6.1
	Tannic Acid	−5.8
	Thymol	−4.7

### 3.6 *In vitro* biocompatibility of HSA/TA coated scaffolds

In addition to preventing bacterial attachment and biofilm formation, antibacterial coatings should be biocompatible and allow optimal implant integration. Standard *in vitro* biocompatibility assessment of biomaterials involves the static seeding of mammalian cells onto the scaffold surface and the subsequent study of the changes in cell viability, metabolic activity, and morphology as a result of their interactions with the biomaterial. This seeding is usually achieved by pipetting small volumes (<50 μL) of highly concentrated single cell suspensions on the biomaterial surface, which can result in poor cell seeding efficiency and heterogeneous cell distribution, as well as impaired functionality due to the lack of cell-to-cell and extracellular matrix (ECM) interactions [63]. In addition, the release of antibacterial agents in small cell suspension volumes may lead to a certain level of toxicity that will not be achieved *in vivo*.

The use of cell sheets offers a more controlled and targeted cell delivery approach as highly dense homogeneous cell layers can be achieved without experiencing cell loss due to inefficient seeding - a well reported drawback for scaffolds with macropores. In addition, these cells are embedded in mature ECM components fully synthesized by them, which creates a physiologically relevant tissue-like structure to test antibacterial coatings. In this study, primary human pre osteoblasts (hPOB) were grown and matured into highly dense mechanically stable cell sheets that were then wrapped on the control and HSA/TA-coated scaffolds. Wrapped scaffolds were cultured for 14 days and changes in cell morphology and cell proliferation were investigated.

**Fig. 6:**
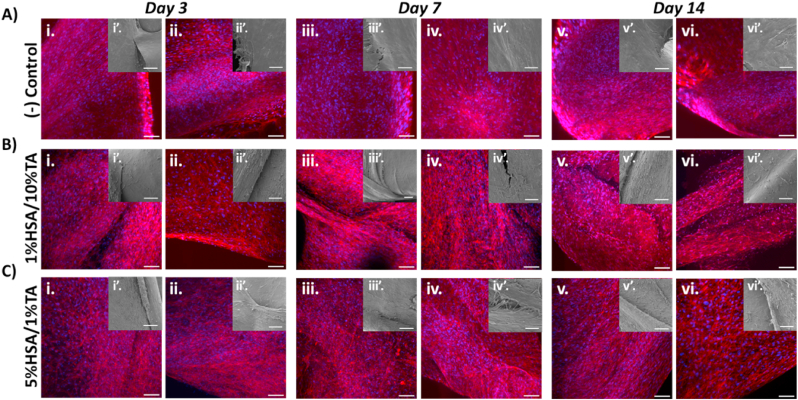
Biocompatibility of HSA/TA-coated scaffolds. Confocal microscopy (DAPI/Phalloidin staining) (i-vi) and SEM (i’-vi’) images of *in vitro* cultured human cell sheets wrapped around A) control, B) 1%HSA/10%TA- and C) 5%HSA/1%TA-coated scaffolds after 3, 7, and 14 days in culture; scale bars: (i-vi) 100 μm, (inlets: i’, iii’, v’) 100 μm, (inlets: ii’, iv’, vi’) 50 μm.

Fig. 6A–C shows the confocal and scanning electron microscopy images of cell sheets wrapped around the untreated and HSA/TA-coated mPCL scaffolds. All scaffolds were fully covered by densely packed cell sheets with significant amounts of secreted mineralized matrix (Fig. S8). Morphological analysis revealed similar features across all groups, with no evidence of cell sheet disruption, suggesting no cytotoxic effects from the HSA/TA coating.

While qualitative *in vitro* biocompatibility assessment through imaging provides valuable insights, it should be complemented with the quantitative evaluation of cell proliferation. Nevertheless, this quantification relies on the use of DNA-binding fluorophores such as CyQuant, PicoGreen and SYBR Green I, that may face challenges when other DNA-binding molecules are present in the substrate or media. Notably, previous studies have proved a strong affinity between TA and DNA [43,44], raising concerns about the reliability of DNA quantification assays in the presence of TA. For instance, TA released from the HSA/TA-coated scaffolds may interact with DNA released from the cell sheets during the digestion process, potentially skewing the assay results (Data not shown). Despite the widely reported binding affinity between TA and DNA, no studies, to the best of our knowledge, have considered the impaired efficacy of DNA-based cell proliferation assays or DNA-binding dyes used in viability assays, such as propidium iodide, when studying TA-based systems. This aspect will be investigated in future studies.

Albumin is a nontoxic, nonimmunogenic, biodegradable and biocompatible protein found in blood plasma, that has been used in drug delivery systems to increase dose bioavailability and efficacy for the treatment of cancer (Abraxane®) and diabetes (Levemir®, Victoza®) [27,28]. In addition, several studies have shown that albumin coating on different substrates supports host cell recruitment and proliferation, resulting in better graft/implant integration and tissue remodeling [[64], [65], [66], [67]]. In comparison, TA is a polyphenol widely found in nature and has several applications in the food, animal, leather, and medical industries. For instance, TA is used as an FDA-approved food flavouring agent at concentrations between 0.001 and 0.04 % [29] and as a feed additive for animals in ranges between 5 and 15 mg/kg of body weight [68]. In addition, TA has been used in different biomedical applications, including wound dressings, medical adhesives, crosslinking agents and drug delivery systems, without any reported cytotoxic effect [42,[69], [70], [71]]. However, a few studies report a possible dose-depending toxicity of TA *in vitro* and *in vivo*. For instance, *in vivo* studies have shown that high concentrations of TA can lead to diminished liver function and severe necrosis when administered orally to mice (2–4.6 g/kg) and rats (2.26 g/kg), and intraperitoneally in sheep (0.1 g/kg) [72,73], possibly due to TA inhibiting digestive enzymes and interfering with the digestion and adsorption of nutrients such as dietary proteins and iron [74], or by causing alterations in the morphology and physiology of liver cells [75], although the exact molecular mechanism is still unknown. Interestingly, several studies have demonstrated that TA selectively inhibits and causes apoptosis to a variety of cancer cells. For instance, TA has been found to induce prostate cancer cell apoptosis by inhibiting signals for lipogenesis and suppressing the lipid metabolism pathway, and inducing endoplasmic reticulum stress by producing and accumulating ROS [76]. Similarly, TA has also been shown to inhibit breast cancer cells by suppressing the cell epidermal growth factor receptor and the natural fatty acid synthase activity, which play an essential role in cancer progression [77,78]. Contrastingly, there is a lack of understanding of the mechanisms involved in TA toxicity to non-cancer cells.

During the cell biocompatibility studies, coated scaffolds acquired a dark colouration when incubated in cell culture media, with a more noticeable effect on the scaffolds coated with higher TA concentration (Figs. S9A and B). This effect was also observed in the solution in which the scaffolds were incubated, with significantly higher absorbance values for the suspensions incubated with 1%HSA/10%TA-coated scaffolds, followed by suspensions incubated with 5%HSA/1%TA-coated scaffolds, in comparison to media incubated with control scaffolds and media alone (Fig. S9C). Brown to dark colourations of TA-based biomaterials have been widely observed in the literature [30,79,80], however, no study, to the best of our knowledge has questioned the nature of this colour change and the effect it may have on the biomaterial properties. This is particularly important as TA is a highly reactive molecule that can form strong complexes with different proteins, phospholipids, metals, and multivalent cations[[81], [82], [83]], some of which can form insoluble aggregates or byproducts with potential cytotoxic properties. For instance, TA has a strong affinity with Fe^3^, forming very stable and insoluble complexes with a characteristic dark colour through redox reaction [81,84]. This complexation has been widely used for different applications in water treatment, magnetic resonance imaging contrast improvement, drug delivery and anticancer nanoparticles, among others [[85], [86], [87], [88]]. While it is unclear whether Fe^3+^/TA complexes are toxic to living organisms, Yang et *al.*, reported a potential adverse effect of these complexes in aquatic ecosystems as a result of dark water formation, an environmental phenomenon in which Fe^3+^ released from soil as a result of abundant rainfall, and TA released from fresh eucalyptus/oak leaves, react and form dark compounds that result in water pollution and fish death [89]. In addition to its Fe^3+^- complexation properties, TA has also been shown to have a strong affinity for metal alkali ions such as K^+^, Na^+^ and Li^+^, as well as divalent cations such as Mg^2+^ and Ca^2+^, which, depending on their concentrations, can lead to the formation of insoluble and colloidally dispersed aggregates at pH ranging from 6.0 to 10.0 [83,[90], [91], [92]]. All these ions, except for lithium, are present in cell culture media as essential inorganic salts to support several cellular physiological processes such as maintenance of the membrane potential, transport of metabolites and amino acids and respiration [93]. Finally, TA is rich in catechol groups that can be easily oxidised into semiquinone and o-benzoquinone under simulated physiological conditions, contributing to a yellow-brown color change [94] widely observed in polyphenol-containing foods and additives [95]. We, therefore, hypothesize that the colour changes on the coated scaffold surface result from multi-molecular complexation between TA and several components of the cell culture media in addition to the natural oxidation of TA compounds in the presence of oxygen.

As a proof of principle, X-ray photoelectron spectroscopy (XPS) spectra of control and coated scaffold surfaces after incubation in cell culture media for one day were acquired (Fig. S9D). All the surfaces showed an increase in the elemental nitrogen content due to the deposition of amino acids and proteins from the cell culture media. In addition, low concentrations of atomic sodium, calcium and phosphorus were detected (Table S1). Even though XPS offers an overview of the chemical composition of the different surfaces, its detection limit does not allow for accurate detection of elements at lower concentrations (0.1–1.0 At%) [96]. Therefore, future work will include the use of more sensitive techniques such as inductively coupled plasma mass spectrometry (ICP-MS) to detect and quantify the different ions present on the surfaces, to further understand the complexes that are formed and their implications on the physicochemical properties of the coatings.

To validate the extensive *in vitro* characterization of the coating's physicochemical stability, antibacterial efficacy, and biocompatibility, we have started studying the HSA/TA coatings in a large preclinical animal model. Preliminary results from this study (Fig. S10) demonstrate our ability to reproducibly apply the coating to significantly larger and geometrically complex mPCL scaffolds (100 mL breast scaffolds), compared to the smaller and simpler lattice scaffolds used in our *in vitro* tests (0.2 mL) (Figs. S10A–C). Importantly, no signs of inflammation or adverse reactions from the coating were observed 3 months post implantation (Figs. S10D–E).

## 4 Conclusion

This study offers a comprehensive examination of the binding mechanism between HSA and TA, as well as the chemical, mechanical, and topographical characteristics of microporous mPCL scaffolds coated with HSA/TA. Notably, our findings demonstrate that the coated scaffolds are effective against microbial growth for up to three days, which significantly reduces the colonization of *S. aureus* and prevents biofilm formation. Moreover, this research suggests that the coating damages the outer cell membrane of *S. aureus*, leading to bacterial killing. Importantly, the HSA/TA coatings did not show any signs of cytotoxicity on *in vitro* cultured human cell sheets. The technology is now at a translational readiness level of 3–4, and we started to study the HSA/TA coating on mPCL breast scaffolds in a large preclinical animal model.

## Ethics approval for *in vitro* biocompatibility study

Human preosteoblasts (hOB) were isolated by explant culture from a male patient undergoing hip arthroplasty following written informed consent (approved by QUT Human Research Ethics Committee approval number 1400001024).

## Funding

This work was supported by the 10.13039/501100021250ARC Industrial Transformation Training Centre for Multiscale 3D Imaging, Modelling, and Manufacturing [IC 180100008], 10.13039/501100000925NHMRC 2008018 – Transformation of the implant paradigm in breast rehabilitation Grant and the Max Planck Queensland Centre.

## CRediT authorship contribution statement

**Silvia Cometta:** Writing – review & editing, Writing – original draft, Visualization, Validation, Software, Resources, Methodology, Investigation, Formal analysis, Data curation, Conceptualization. **Bogdan C. Donose:** Writing – review & editing, Methodology, Investigation, Data curation. **Alfredo Juárez-Saldivar:** Writing – review & editing, Software, Investigation, Data curation. **Akhilandeshwari Ravichandran:** Writing – review & editing, Methodology, Investigation, Data curation. **Yanan Xu:** Writing – review & editing, Methodology, Investigation, Data curation. **Nathalie Bock:** Writing – review & editing, Investigation. **Tim R. Dargaville:** Writing – review & editing, Supervision, Investigation, Formal analysis. **Aleksandar D. Rakić:** Writing – review & editing, Methodology, Investigation. **Dietmar W. Hutmacher:** Writing – review & editing, Writing – original draft, Supervision, Resources, Methodology, Investigation, Funding acquisition, Formal analysis, Conceptualization.

## Declaration of competing interest

The authors declare that they have no known competing financial interests or personal relationships that could have appeared to influence the work reported in this paper.
