# Supplementary material for: Unravelling the physicochemical and antimicrobial mechanisms of human serum albumin/tannic acid coatings for medical-grade polycaprolactone scaffolds
Source: Bioact Mater. 2024 Aug 28;42:68–84. doi: 10.1016/j.bioactmat.2024.08.023 (PMC11399811; doi:10.1016/j.bioactmat.2024.08.023)

# **Supplementary Information**


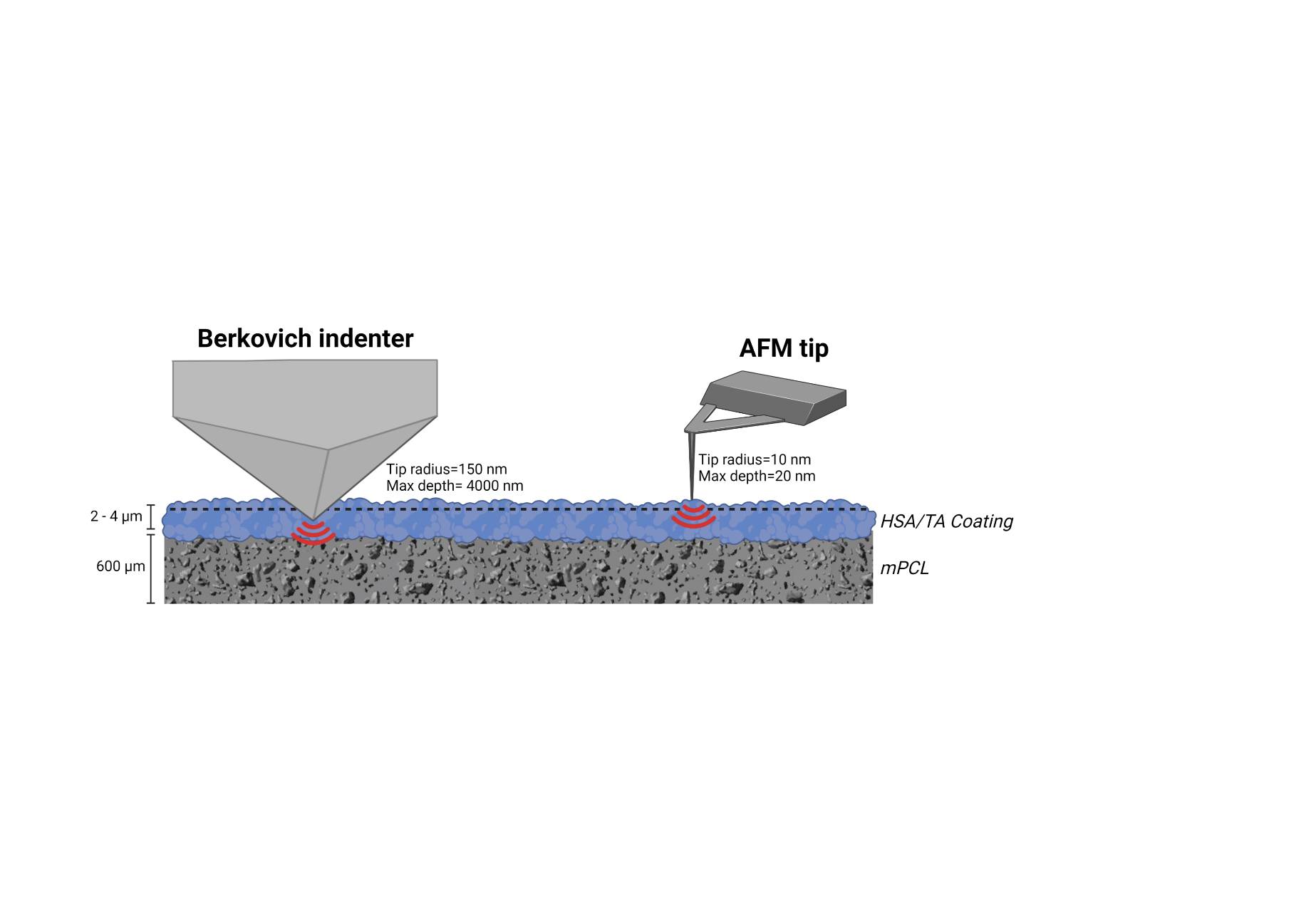


**Figure S1.** Schematic representation of nanoindentation and AFM viscoelasticity mapping highlighting their key features and differences in scale (created with Biorender).


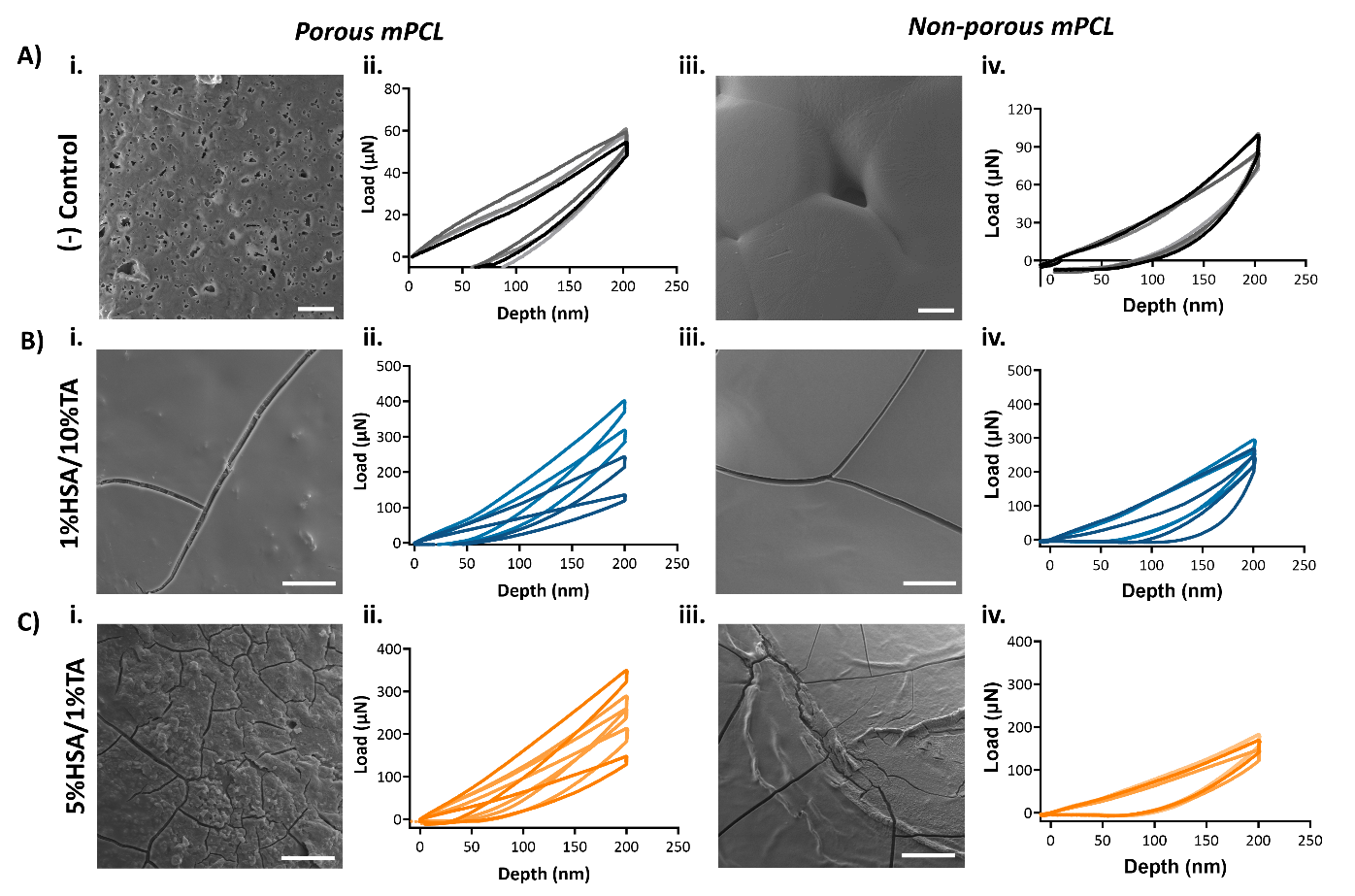
**Figure S2.** Mechanical characterization of coated scaffolds at the micro-scale. Comparison of SEM images (i,iii) and loading curves at a maximum indentation depth of 200 nm (ii, iv) of microporous (left panel) and non-porous (right panel)A) unmodified and B) 1% HSA/10%TA- and C) 5%HSA/1%TA- coated mPCL scaffolds. Scale bars: 20 µm


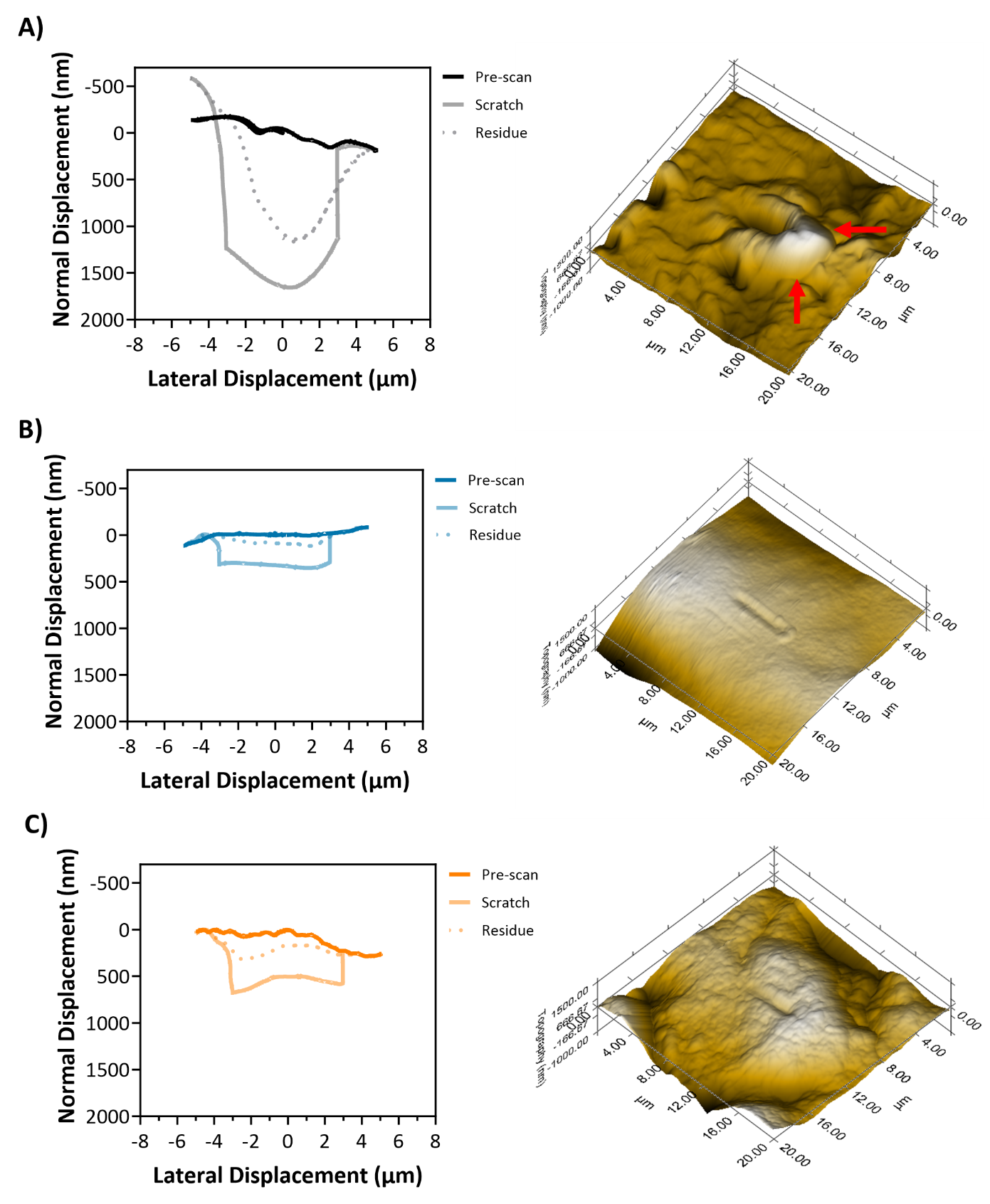


**Figure S3.** Nanoscratch test results. Normal displacement vs lateral displacement before (pre scan), during (scratch) and after scratch (residue), as well as 20 µm x 20 µm surface area reconstruction of nanoscratsched A) unmodified and B) 1%HSA/10%TA- and C) 5%HSA/1%TA-coated mPCL surfaces. Red arrows highlight material pile-up around the indentation groove.


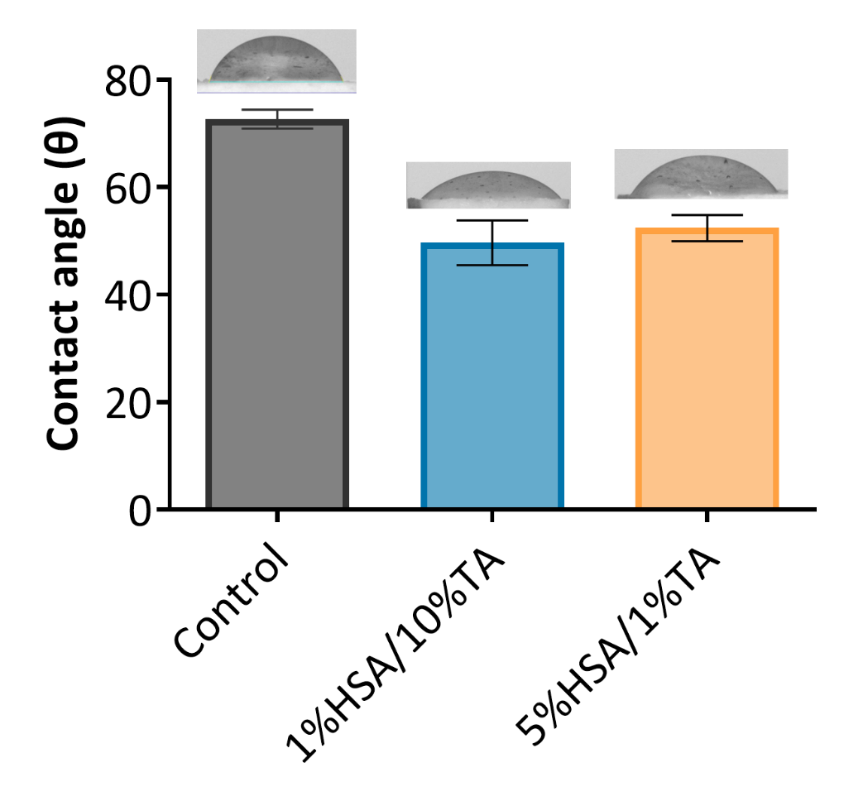


**Figure S4.** Surface characterization after HSA/TA immobilization. Difference in contact angle among all the surfaces.


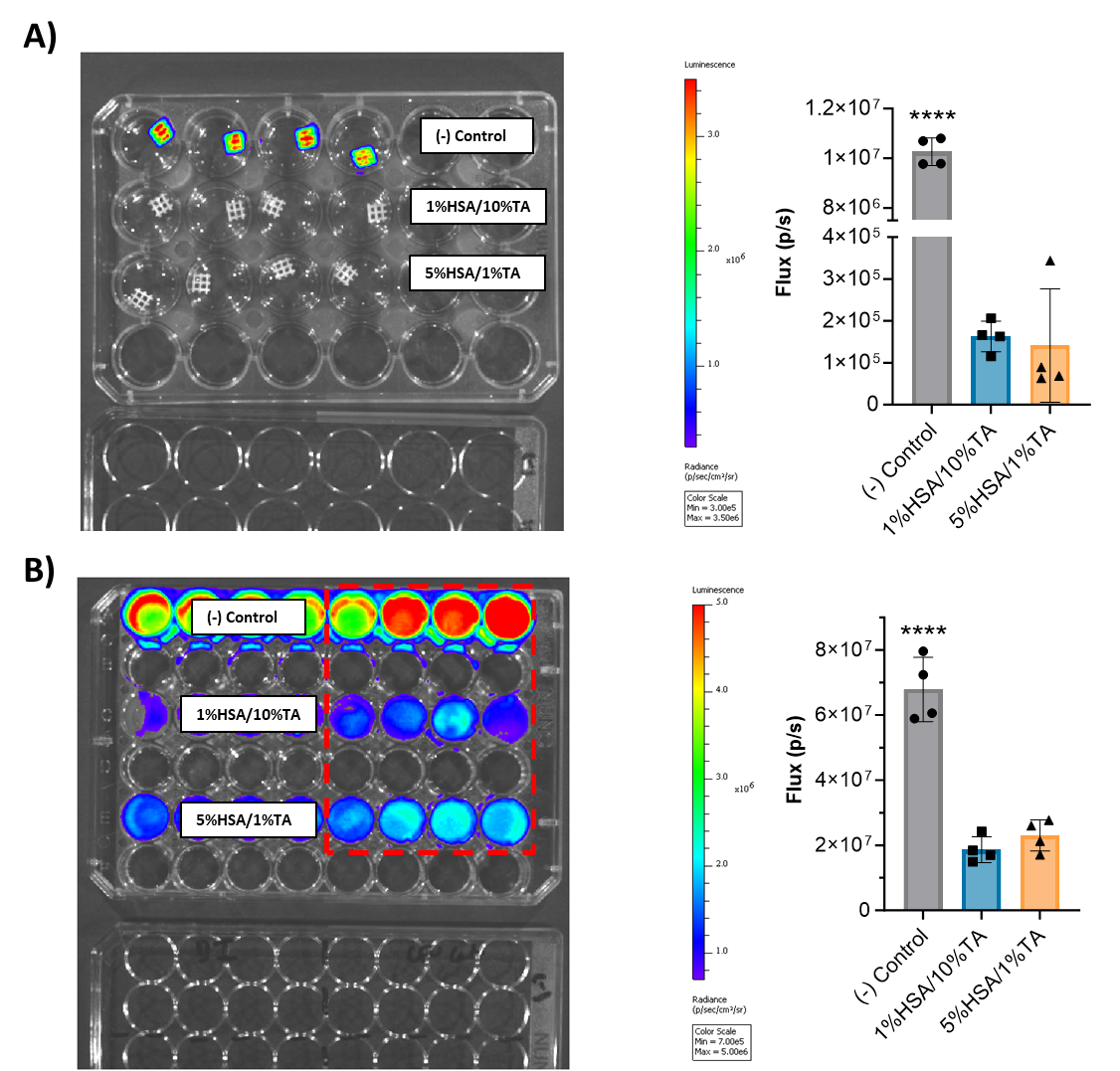


**Figure S5.** Antimicrobial activity of HSA/TA-coated scaffolds against *S. aureus* Xen 36 after one day in culture. A) Bioluminescence imaging of scaffolds exposed to *S. aureus* Xen 36 evidencing significant bacteria attachment on unmodified surfaces, in comparison to coated scaffolds in which bioluminescence signal is close to the lower detection limit. The bar graph shows the difference in total photon flux (p/s) of control and modified scaffolds. B) Bioluminescence imaging of bacteria suspension in which coated and unmodified scaffolds were incubated overnight, evidencing the bactericidal effect of the coating components released into the media. The bar graph shows the difference in total photon flux (p/s) of suspensions exposed to control and modified scaffolds. All images from the three groups were taken at the same time and at are displayed at the same intensity scale. Data shown as mean ± SD, ** p*<0.05; *** p*<0.01; **** p*<0.001;***** p*<0.0001, (n=4).


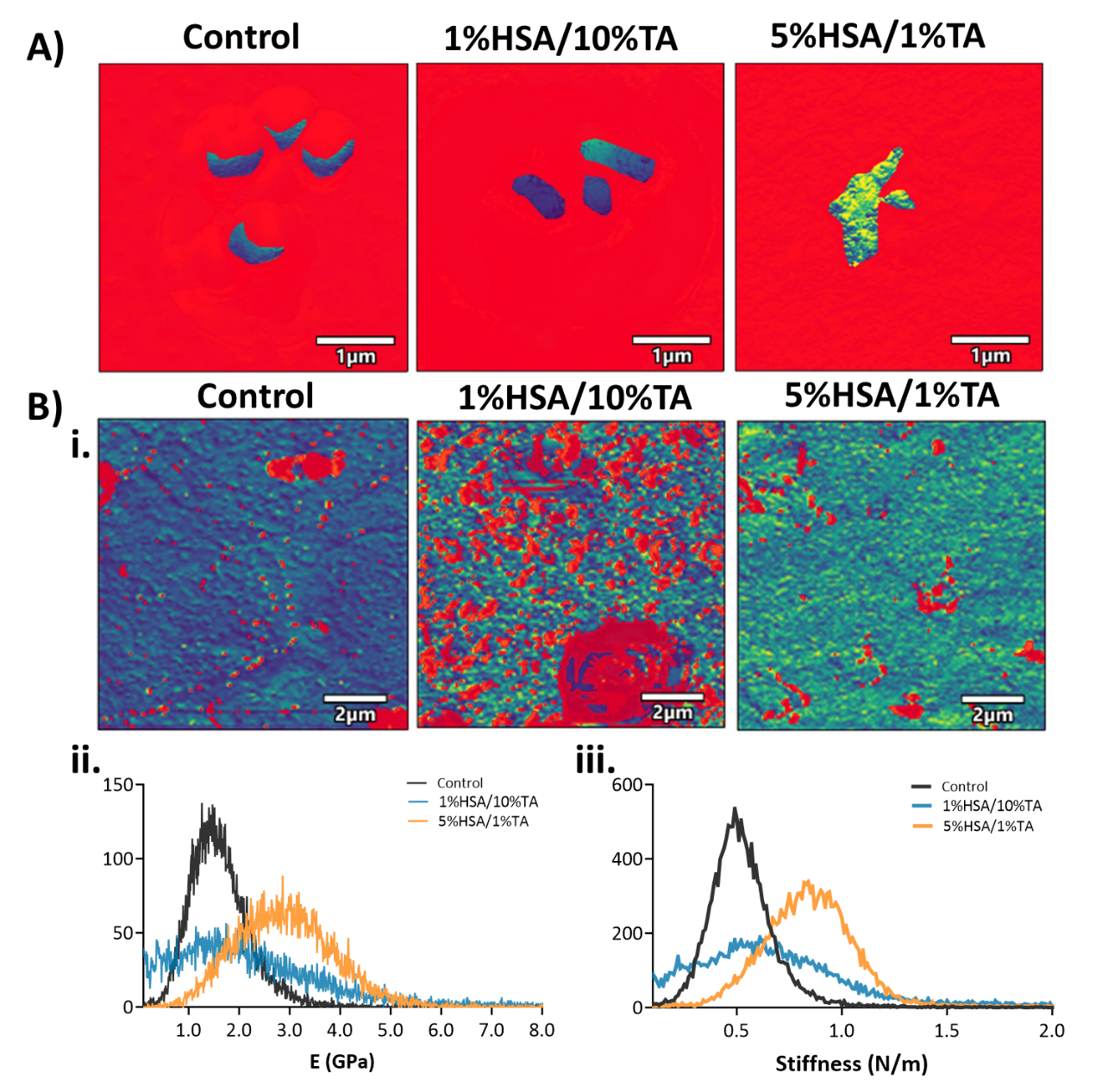


**Figure S6.** Masked surface areas for the determination of the mechanical properties of A) Bacteria and B) the substrate on control and HSA/TA-coated surfaces. Histograms of iv. Young’s moduli and v. stiffness for i. control ii. 1%HSA/10%TA and iii. 5%HSA/1%TA scaffolds. Histograms include the measurement of three different scaffolds.


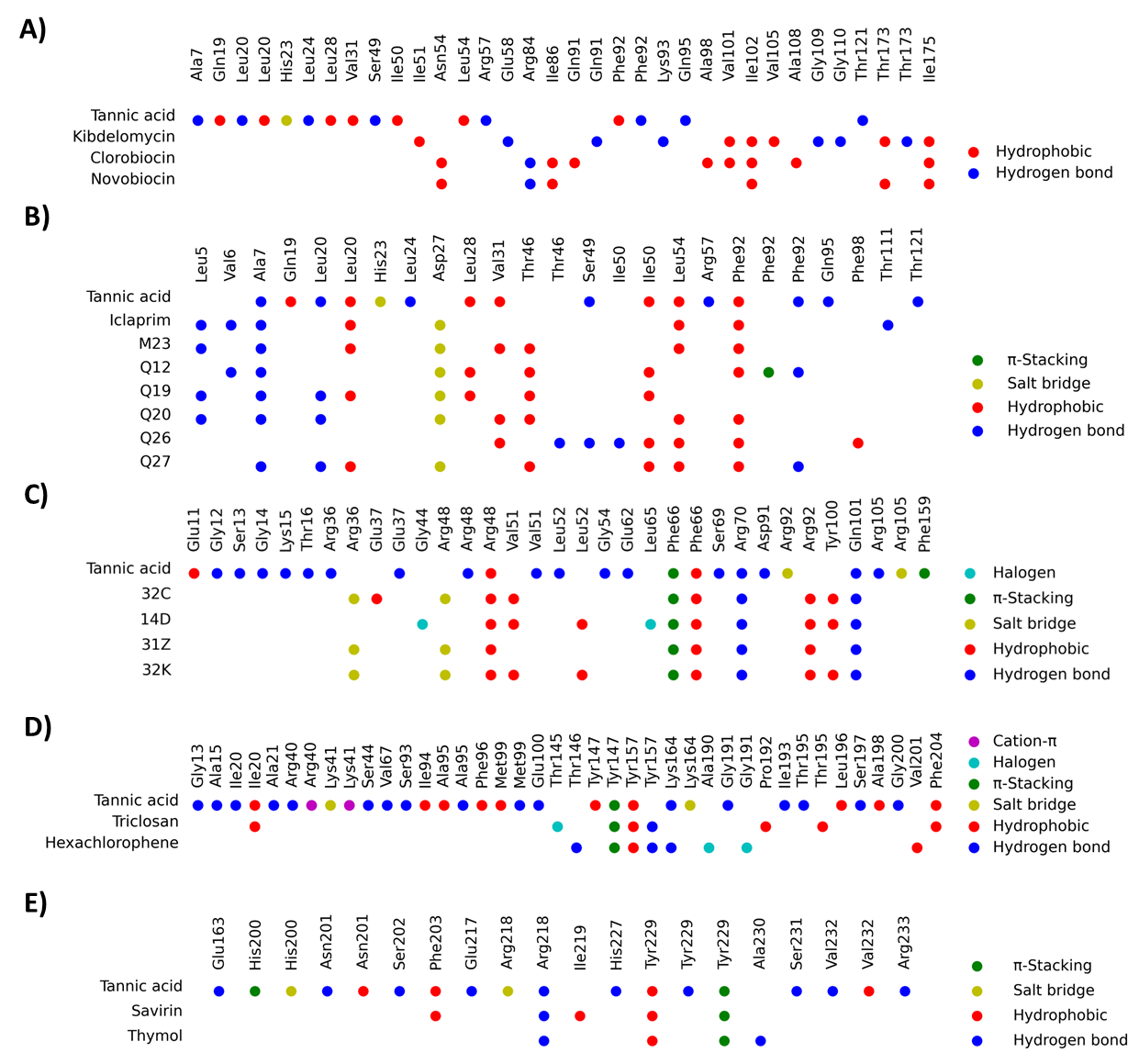


**Figure S7.** Comparisons of interaction profiles between TA and known inhibitors with *S. aureus* targets A) DNA gyrase subunit B, B) Dihydrofolate reductase, C) Thymidylate kinase, D) Enoyl-[acyl-carrier-protein] reductase and E) Accessory gene regulator quorum sensing system.


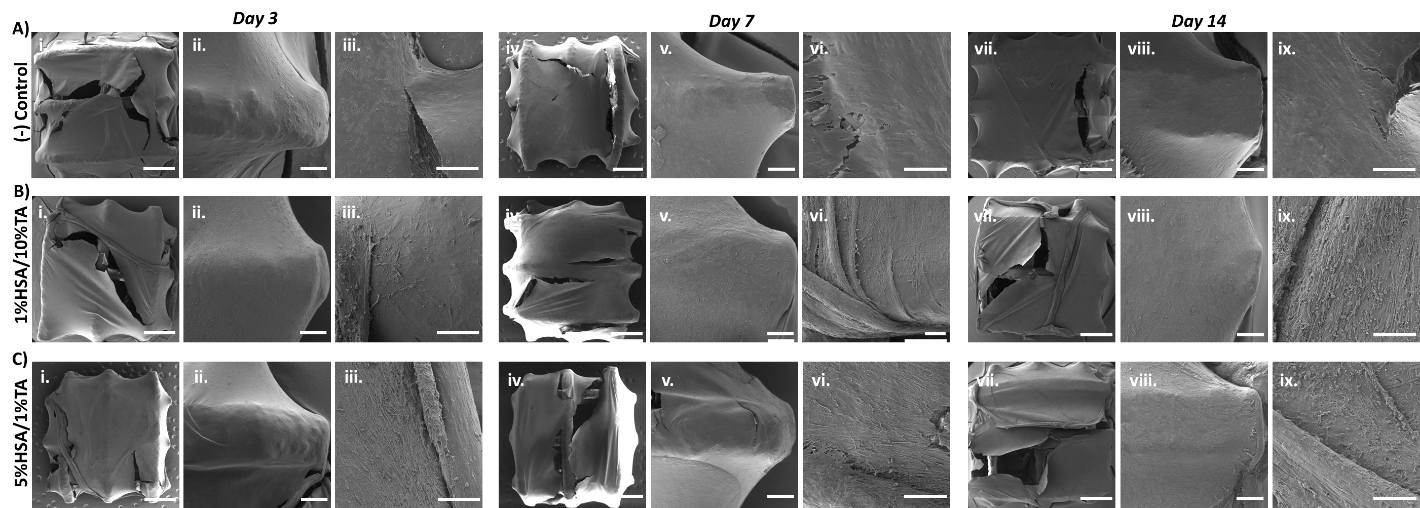


**Figure S8.** Biocompatibility of HSA/TA-coated scaffolds. SEM images of *in vitro* cultured human cell sheets wrapped around A) control, B) 1%HSA/10%TA- and C) 5%HSA/1%TA-coated scaffolds after 3, 7, and 14 days in culture; scale bars: (i, iv, vii) 1 mm, (ii, v, viii) 200 µm, (iii, vi, ix) 100 µm.


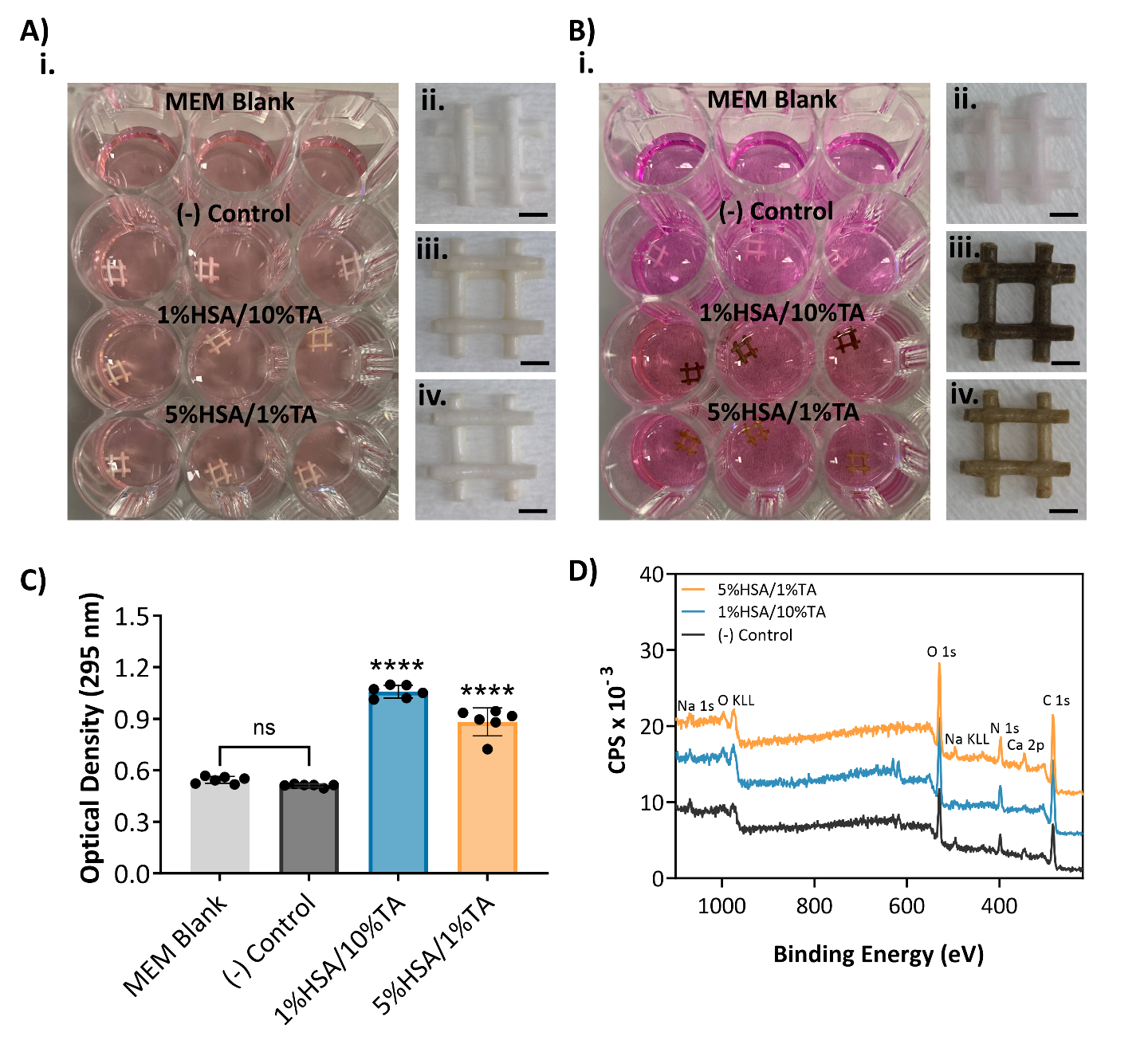


**Figure S9.** Dark coloration of scaffolds due to possible complexation with iron ions present in the cell culture media and that could be toxic. A) scaffolds before and B) after incubation in DMEM for one day. C) Optical density at a wavelength of 295 nm that allows to detect differences because of the release of TA and possible reaction with the media and D) XPS wide spectra of scaffolds after incubation in media. Data shown as mean ± SD, ** p*<0.05; *** p*<0.01; **** p*<0.001;***** p*<0.0001, (n=3).


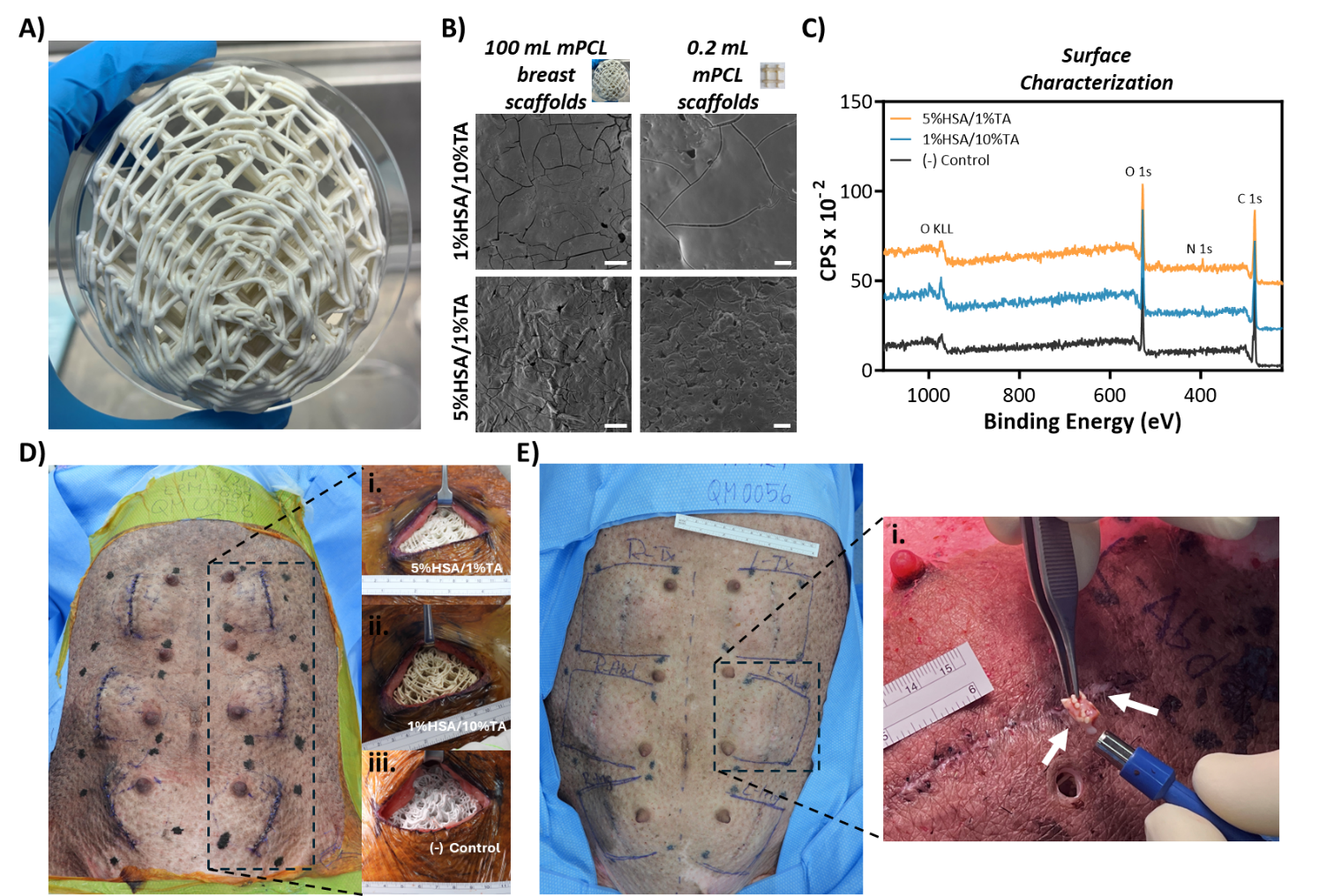


**Figure S10.** Preliminary results from the study of HSA/TA-coated mPCL breast scaffolds in an *in vivo* pig model. A) 100 mL mPCL breast scaffolds coated with 1%HSA/10%TA. B) SEM images comparing HSA/TA-coated 100 mL mPCL breast scaffolds used *in vivo* and lattice mPCL scaffolds (0.2 mL) used *in vitro*, showing comparable coating morphologies. Scale bars: 20 µm C) XPS wide spectra of unmodified and HSA/TA-coated mPCL breast scaffolds, demonstrating similar surface chemistry between the coated large-scale and small-scale scaffolds, highlighting reproducible homogeneous coating of HSA/TA on geometrically different mPCL surfaces. D) Postoperative photo illustrating six implantation sites with corresponding incisions for each subglandular pocket, including intraoperative images of implants coated with i. 5%HSA/1%TA, and ii. 1%HSA/10%TA, and iii. unmodified mPCL breast scaffolds. E) Three-month postoperative wound review showing well-healed wounds without infection or inflammation, with a biopsy from the 1%HSA/10% TA-coated breast scaffold (white arrows).

**Table S1.** Elemental composition of unmodified and HSA/TA-coated scaffolds after incubation in cell culture for 24h.


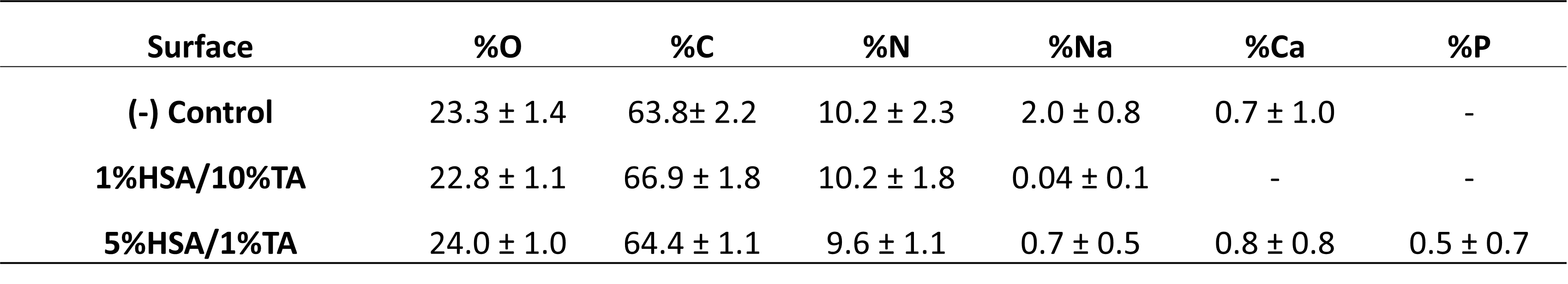

Supplement: Multimedia component 1 [file mmc1.docx]
